# Enhancing portfolio management using artificial intelligence: literature review

**DOI:** 10.3389/frai.2024.1371502

**Published:** 2024-04-08

**Authors:** Kristina Sutiene, Peter Schwendner, Ciprian Sipos, Luis Lorenzo, Miroslav Mirchev, Petre Lameski, Audrius Kabasinskas, Chemseddine Tidjani, Belma Ozturkkal, Jurgita Cerneviciene

**Affiliations:** ^1^Department of Mathematical Modeling, Kaunas University of Technology, Kaunas, Lithuania; ^2^School of Management and Law, Institute of Wealth and Asset Management, Zurich University of Applied Sciences, Winterthur, Switzerland; ^3^Department of Economics and Modelling, West University of Timisoara, Timisoara, Romania; ^4^Faculty of Statistic Studies, Complutense University of Madrid, Madrid, Spain; ^5^Faculty of Computer Science and Engineering, Ss. Cyril and Methodius University in Skopje, Skopje, North Macedonia; ^6^Complexity Science Hub Vienna, Vienna, Austria; ^7^Division of Firms and Industrial Economics, Research Center in Applied Economics for Development, Algiers, Algeria; ^8^Department of International Trade and Finance, Kadir Has University, Istanbul, Türkiye

**Keywords:** portfolio, asset allocation, artificial intelligence, machine learning, optimization, rebalancing, explainability, regulation

## Abstract

Building an investment portfolio is a problem that numerous researchers have addressed for many years. The key goal has always been to balance risk and reward by optimally allocating assets such as stocks, bonds, and cash. In general, the portfolio management process is based on three steps: planning, execution, and feedback, each of which has its objectives and methods to be employed. Starting from Markowitz's mean-variance portfolio theory, different frameworks have been widely accepted, which considerably renewed how asset allocation is being solved. Recent advances in artificial intelligence provide methodological and technological capabilities to solve highly complex problems, and investment portfolio is no exception. For this reason, the paper reviews the current state-of-the-art approaches by answering the core question of how artificial intelligence is transforming portfolio management steps. Moreover, as the use of artificial intelligence in finance is challenged by transparency, fairness and explainability requirements, the case study of *post-hoc* explanations for asset allocation is demonstrated. Finally, we discuss recent regulatory developments in the European investment business and highlight specific aspects of this business where explainable artificial intelligence could advance transparency of the investment process.

## 1 Introduction

**Figure 1:**
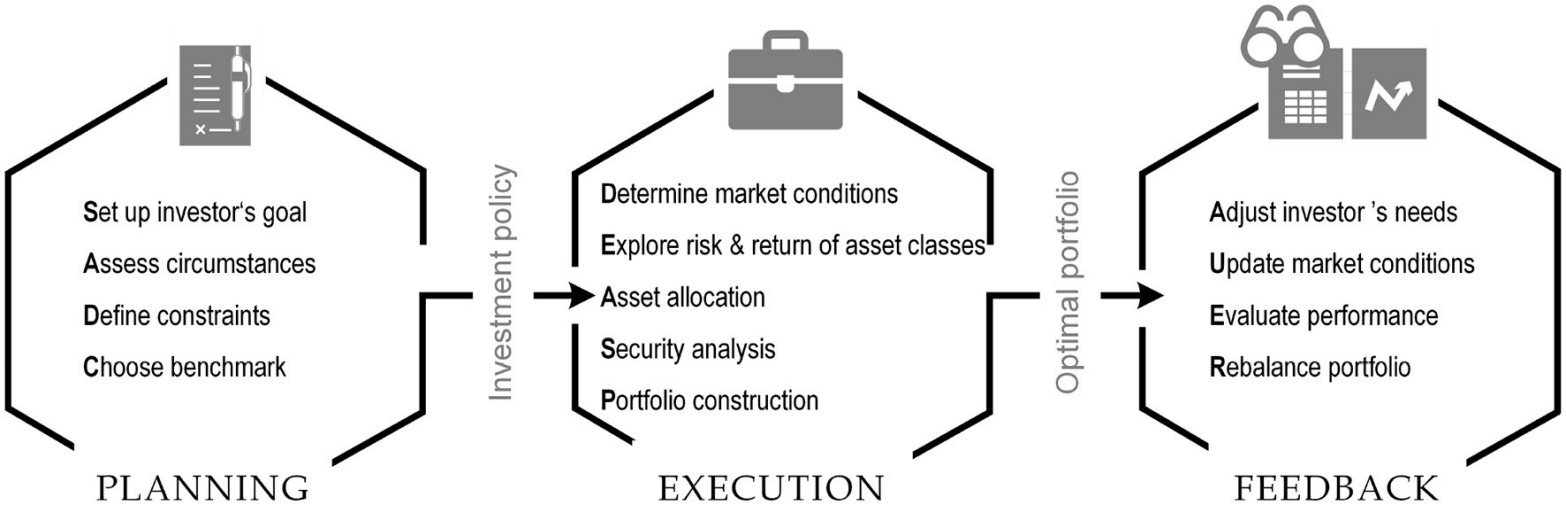
Portfolio management process.

Portfolio management is a continuous process of creating portfolios based on an investor's preferred level of risk and reward and then adjusting it over time to maximize returns. This process includes three subsequent layers, namely planning, execution, and feedback (see Figure 1) (Baker and Filbeck, 2013). The first layer of the process is the planning layer. The asset owner—an institutional client like a pension fund or a wealth management client—mandates an asset manager to manage a specific portfolio according to an investment policy. The investment policy defines this mandate. It contains the client's needs, circumstances, and constraints to achieve a particular reward goal at a given risk level. Strategic asset allocation (SAA) is part of this investment policy. Typically, the SAA is defined as upper and lower boundaries for the asset class allocation. The risk tolerance and risk capacity also need to be defined. The second layer of the portfolio management process is the execution layer. The execution starts with determining the overall macroeconomic conditions across countries and asset classes, exploring the risk-and-return characteristics of asset classes. This analysis determines the capital allocation across countries and asset classes (“tactical asset allocation”). Security analysis enables the cross-sectional selection of single securities within each asset class to construct the overall portfolio and execute the necessary trades. Finally, after the portfolio experienced the market dynamics of an investment period, the feedback layer evaluates past performance, updates the market conditions, checks if the investment policy still holds or needs to be adjusted, and finally rebalances the portfolio (Bailey et al., 2007; Horn and Oehler, 2020).

Portfolio construction has been a significant task since 1952 when Markowitz introduced the mean-variance model. This model inspired many researchers, leading to numerous research papers proposing its extensions to overcome the shortcomings that neglected real-life problems. However, the continuously changing market environment, globalization, and integration of financial markets have generated new challenges in portfolio management, such as high systemic risk, spillover effect, contagion channels, and geopolitics risk.

In recent years, artificial intelligence (AI) has disrupted most industries, including the financial sector. AI techniques can contribute to portfolio management in many ways, improving the shortcomings of classical portfolio construction techniques and extending the opportunities to generate additional alpha. For instance, machine learning (ML) can create systems that learn from experience and be used for asset price prediction. Reinforcement learning (RL) is one of the most promising tools for developing a sequential and dynamic portfolio optimization theory. Text mining and sentiment analysis can enhance portfolio management with fresh news from the market. Dimensionality reduction methods can detect latent factors of a broad range of asset prices, which improves the construction of a well-diversified portfolio. Deep learning can optimize an investment portfolio directly or establish a portfolio that mimics an index with a small set of assets.

AI can produce better asset return and risk estimates and solve portfolio optimization problems under complex constraints, resulting in better out-of-sample AI-based portfolio performance than traditional approaches. From a technical point of view, the key players in the financial sector are embracing AI as a tool for automating and enhancing operational efficiency, processing vast amounts of data, improving risk management, and suggesting solutions that better suit investors' needs and accommodate risk. On the other hand, AI-based portfolio management often means that the decision is generated from a black-box model instead of mathematical equations trained on some database. This raises additional challenges in explaining and interpreting the decisions made by AI to earn the trust of various stakeholders, such as shareholders, investors, or portfolio managers.

The principal goal is to identify and evaluate published papers that propose AI-based methods for portfolio construction. To accomplish this, we focus on key considerations within this field, focusing on three main portfolio management steps (see Figure 1). The strengths and limitations of popular approaches used for portfolio construction are reviewed during the analysis, addressing these considerations. Moreover, to emphasize the need for transparency and fairness of decisions, laminable artificial intelligence (XAI) area approaches are briefly reviewed, and a case study of *post-hoc* explanations for portfolio construction is presented. Notably, the current review extends the most recent survey (Bartram et al., 2021) that focused on ML approaches and empirical results relevant to active portfolio management. In their paper, the authors considered using ML for signal generation, NLP applications, and several applications of reinforcement learning. Additionally, active AI-driven ETFs could be an excellent example of growing investor interest. However, the questions concerning portfolio optimization, portfolio evaluation and rebalancing, and *post-hoc* explainability of portfolio performance have not been addressed. Another review (Bartram et al., 2020) recently published by CFA mainly focuses on AI applications for asset classification and forecasting. Additionally, the use of NLP for automatic analysis of corporate annual reports, news articles and Twitter posts is presented. Examples of evolutionary algorithms and artificial neural networks are provided for portfolio optimization tasks, accommodating the flexibility to solve complex multi-objective asset allocation problems. Another example of a literature review (Nuzzo and Morone, 2017) outlined the main advances in using experimental techniques to study financial markets. Their work is not directly related to portfolio management but presents the relevant issues about information release and market structure, explores some stylized facts of the distribution of returns, and considers the role of market institutions in trading activity. Comparatively, the extensions of a mean-variance framework have long been an area of particular interest to many researchers, based on which some reviews (Elton and Gruber, 1997; Steinbach, 2001) have been published.

## 2 Investment portfolio management in a nutshell

**Figure 2:**
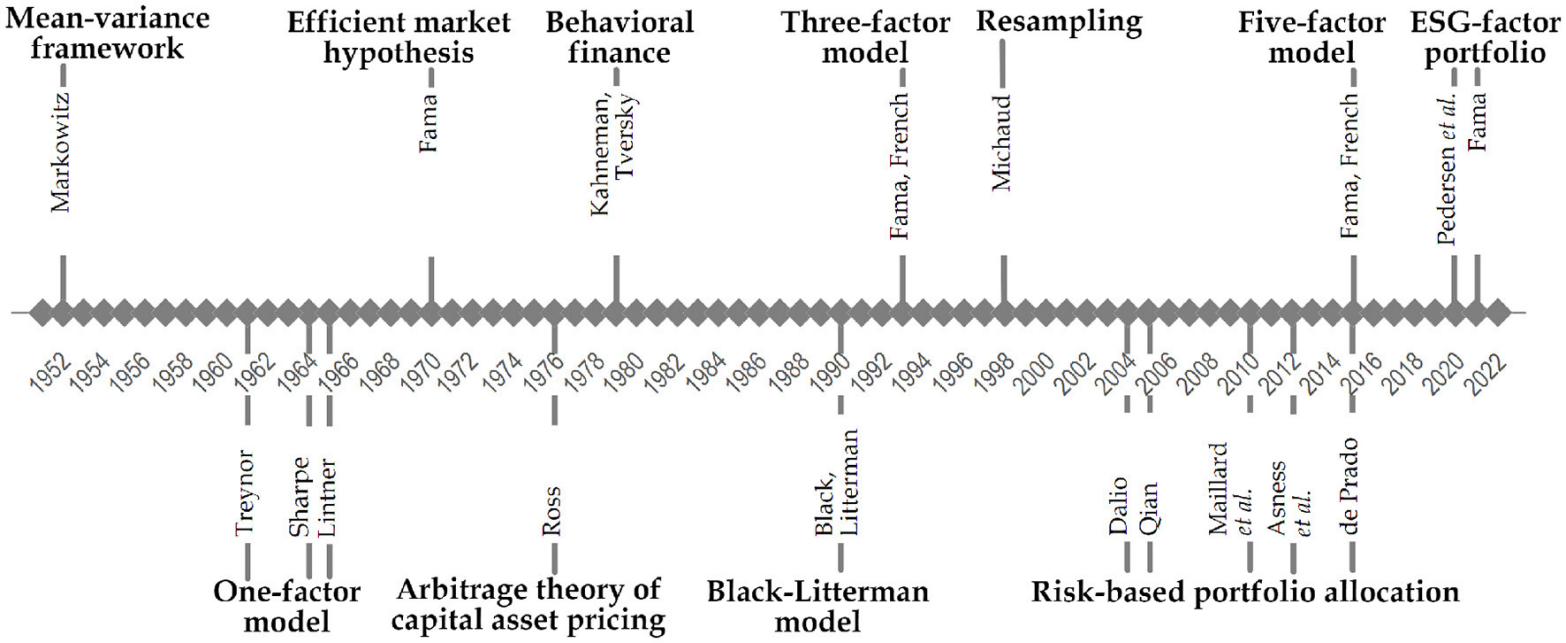
Advancements in investment portfolio management.

Hally, we could distinguish some famous frameworks and theories that remarkably impacted the way of thinking and modeling how to construct an investment portfolio and initiated the literature strands accordingly (see Figure 2).

Markowitz (1952, 1959) marks the birth of modern portfolio theory (MPT) by introducing the mean-variance efficient frontier framework. As the name suggests, the mean and variance have been employed to measure a portfolio's expected return and risk. The main message was that the investments should not be selected by combining multiple individual securities with preferable risk and return characteristics but by determining how they contribute to the overall portfolio. The efficient frontier concept was formulated based on two distributional measures, namely mean and variance, from which the investor could choose the preferred asset allocation. Notably, the derivation of the mean-variance framework was based on several essential assumptions (Elton and Gruber, 1997; Wilford, 2012). Despite criticism, the mean-variance theory remains crucial. Like other breakthroughs, it has been extended in various directions.

Inspired by Markowitz work, Treynor (1962), Sharpe (1964), and Lintner (1965) independently introduced a factor model, named as Capital Asset Pricing Model (CAPM). Specifically, CAPM is the instance of the one-factor model, which describes the relation between systematic risk and expected returns. Technically, CAPM decomposes an asset's return into factors common to all assets and factors specific to a particular asset. However, one factor is not enough to quantify risk and returns adequately. This resulted in so-called multi-factor models generalized by Ross (1976); Roll and Ross (1980), known as Arbitrage Pricing Theory (APT). The primary difference between CAPM and APT is how a systematic investment risk is defined. CAPM includes a single, market-wide risk factor, while APT advocates several factors which capture market-wide risks.

The efficient market hypothesis (EMH) is one of the milestones in the MPT development (Vamvakaris et al., 2017). Its roots go back to the period of 1963–1965, with the appearance of some works published by Fama (1963), Fama (1965), and Samuelson (1965). According to the Delce (2019) and Lo (2017b), Fama suggested the concept of an efficient market known for its best formulation: “A market in which prices always fully reflect available information is called efficient” (Fama, 1970). Comparatively, Samuelson's contribution to the development of EMH is less well-known, but his role is no less important as he provided a solid theoretical basis for this hypothesis. Since then, many studies have been published on examining whether the EMH is valid in different markets, for example, stock market (Lee et al., 2010; Sánchez-Granero et al., 2020), energy market (Lee and Lee, 2009; Liu et al., 2020), currency market (Potì et al., 2020). The idea behind testing EMH is to measure whether a random market walk is related to price predictability. For this purpose, different kinds of tests for market efficiency have been proposed addressing the concept of random walk (Frunza, 2016). However, there exists enough evidence to infer that the existence of an efficient market seems to be a utopia in practice. Instead, it is more realistic to anticipate relative efficiency, identifying periods with varying degrees of efficiency influenced by changing market conditions over time (Campbell et al., 1998; Kim et al., 2011; Alvarez-Ramirez et al., 2012).

The main alternative to CAPM is the three-factor model (Fama and French, 1993), which become widely used by academics and practitioners. This model included two additional factors, proxy size and value, for estimating cross-sectional equity returns. Two decades later, this model has been extended to the five-factor model (Fama and French, 2015), which includes profitability and investment of the firm in addition to market factor, firm size and value, aiming to describe better the variation in equity prices that the three-factor model does not capture. Over a considerable time, these models have been extensively tested empirically by numerous studies aiming to adequately price the equity returns in both developed and emerging markets (Kubota and Takehara, 2018; Lalwani and Chakraborty, 2019; Mosoeu and Kodongo, 2020). The evidence shows, for example, (Mohanty, 2019), that each market is unique in its composition and trend even over a long time horizon, and hence, a generalized asset pricing model cannot be adopted across all the markets.

The other stream entails the problems arising from the assumptions of “homo economicus”. The field of behavioral finance occurred in the late 1970s as a response to emerging failures of the core pricing models to explain anomalies in financial markets (Kahneman and Tversky, 1979; Kumar, 2016). Behavioral finance indicates that when making decisions like investing, people are not nearly as rational as traditional finance theory assumes. Similarly, Shiller (2003) provides an insight into the changes in the approaches and focuses on the weaknesses of the efficient market hypothesis, trying to explain the financial markets better by understanding and incorporating the inefficiencies and biases in the models. Later, Thaler (1999) extends the idea of behavioral finance of incorporating psychological components to be included in all financial models in the future, as otherwise would be irrational. Lo (2004) and Lo (2017a) suggest that behavioral aspects in the portfolio decision-making process align with an evolutionary model with a perspective of adaptation, and this new approach combining economy and psychology is called the “Adaptive Market Hypothesis”.

In the past decade, there has been a surge in work exploring AI applications across various domains, including investment portfolio management. However, there is no widely acknowledged what could have been the first attempts of AI employment for asset allocation tasks. Considering the current taxonomy of AI approaches, for example, (Schmid et al., 2021), we believe that the Black-Litterman model (Black and Litterman, 1991) could be a potential candidate. In particular, their model suggests a framework for combining market equilibrium information with subjective investors' views by exploiting a Bayesian methodology. The computational evidence shows that the Black-Litterman model produces more stable and better-diversified portfolios than those constructed under Markowitz framework (Rebonato and Denev, 2014).

An alternative to address estimation uncertainty parametrically is Monte Carlo resampling (Michaud, 1998), a procedure to determine portfolio weights as average weights from MPT results derived from bootstrapped market returns. In institutional active portfolio management, leveraged risk-based multi-asset allocations without return estimations are popular, namely Risk Parity (Qian, 2005; López de Prado, 2016; Dalio, 2004), Equal Risk Contribution (Maillard et al., 2010), and inverse-volatility weighting (Asness et al., 2012). A significant milestone is the Hierarchical Risk Parity (HRP) approach (López de Prado, 2016) aimed to improve the robustness of Risk Parity schemes in markets with fluctuating covariances. In the first step, HRP sorts markets via a single-linkage clustering procedure. In the second step, market weights are allocated using a bisection of the covariance matrix.

Environmental, social and governance (ESG) factors and socially responsible investments (SRI) examine how conscious the companies invested are in these areas. Another angle of portfolio optimization in recent years is ESG and SRI evaluation. They become more critical and create a new perspective for investors as the maximization of shareholder value is changing to the maximization of welfare (Fama, 2021). For example, a recent paper Pedersen et al. (2020) designed an ESG-efficient frontier with the highest Sharpe ratio for the ESG-adjusted CAPM, where the choice may lead to a positive, negative or neutral outcome.

## 3 Artificial intelligence approaches for signal generation

**Figure 3:**
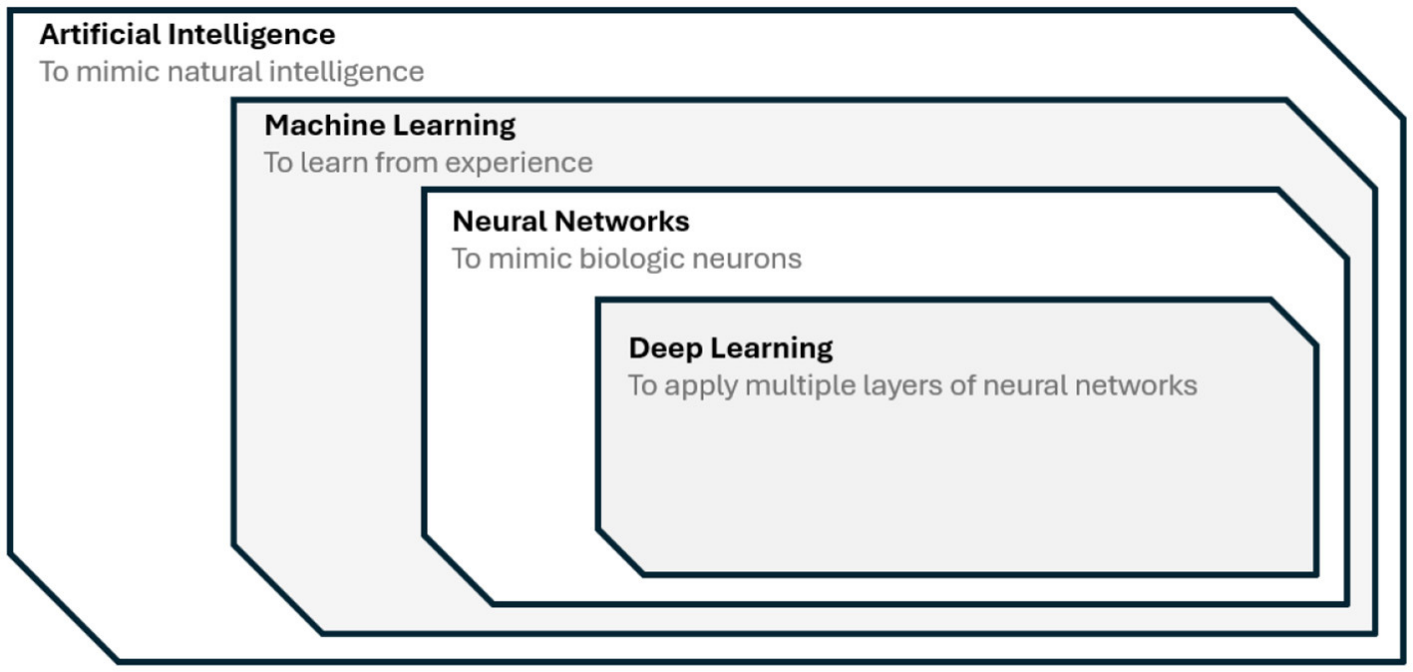
AI, ML, NN, and DL relationship diagram.

AI techniques can be considered decision tools with a straightforward application to the different stages of portfolio execution (see Figure 3). The ability to describe underlying market structures, process vast amounts of structural and non-structural information, or capture the non-linearity between different variables makes AI a key role in handling market complexity. AI tools guide the portfolio manager through the entire process, from visualizing the market to identifying assets, constructing the portfolio, executing trades, and interpreting results. This contributes toward achieving trust in AI-driven portfolio management systems. This section introduces AI techniques beneficial for various subtasks in portfolio management, contributing to trust in AI-driven systems.

### 3.1 High-dimensional forecasting and predictors selection based on linear models

Two conventional dimensionality reduction techniques that help the portfolio manager tackle the market complexity are Principal Component Regression (PCR) and Partial Least Square (PLS), regression-based procedures designed to forecast time series parsimoniously. The first is a two-step procedure that involves constructing the principal components using Principal Components Analysis (PCA) and then using these components as the predictors explaining most of the variance in a linear regression model. The first principal component can be taken as a proxy of the market factor. The study in Stock and Watson (2002) provides a notable example of simplifying a high-dimensional forecasting problem with numerous predictors by modeling time series variability using a small number of latent factors. Feasible forecasts are asymptotically efficient, and, more importantly, the estimated factors remain consistent, even in the presence of time variation in the factor model. The link between portfolio optimization models and PCA is straightforward, as explained in Meucci (2009); Partovi and Caputo (2004). The more natural choice of uncorrelated risk for a portfolio is by a PCA decomposition of the return covariance Σ, i.e.,


(1) $$\begin{array}{l} E^{\prime }\Sigma \text{E}\equiv \Lambda , \end{array}$$

where the diagonal matrix Λ≡*diag*(λ_1_, ..., λ_*N*_) contains the eigenvalues of Σ, sorted in decreasing order. In this way, the complexity of portfolio selection is reduced if there are no correlations among the assets.

Comparatively, PLS regression reduces dimensionality by incorporating the forecasting objective or response. The linear combinations maximize the covariance between the target variable and each standard component obtained from the predictors (Groen and Kapetanios, 2016). Kelly and Pruitt (2013) is one of the first attempts to apply PLS regression to finance. In Kelly and Pruitt (2015), the three-pass regression filter (3PRF) was proposed, which has been proven to be consistent for the infeasible best forecast when both the time dimension and cross-section dimension become large. Unlike PLS, the 3PRF enables the selection of additional disciplining variables based on economic theory.

PCR and PLS are techniques that merge the set of predictors from dimension *D* to a much smaller number of *L* linear combinations. Comparatively, Ridge, LASSO and Elastic net methods focus more on shrinkage, moving the model coefficients to zero. Ridge penalizes the square sum of coefficients called *l2*, reducing the variance compared with Ordinary Least Square (OLS). LASSO regularization penalizes the absolute sum of coefficients called *l1* shrunk toward zero, achieving a selection of the predictors, which outperforms OLS as well (Messmer and Audrino, 2020). Elastic net includes a regularization that combines *l1* and *l2*, handling the weight of each by a hyper-parameter. Specifically, LASSO, a form of regularized regression, combines variable selection and regularization to improve prediction accuracy. It automatically selects the most predictive input factors from a set (Feng et al., 2017; Freyberger et al., 2018), enabling the exploration of lead-lag relationships between asset groups. This approach is crucial in determining influential predictors, such as industry or market output, preventing overfitting, and controlling model complexity in machine learning methods (Li, 2015; Gu et al., 2020).

Table 1 gives good examples of selecting significant predictors.

**Table 1:** Forecasting with a high number of potential predictors.

**Application purpose**	**Method**	**Description**	**References**
Combined index	Dynamic factor model	Development of new indexes to represent leading and coincident economic indicators	Stock and Watson, 1989, 1998
Feature selection	Double-selection estimation procedure	Framework for systematically evaluating the contribution of individual factors relative to existing factors	Feng et al., 2020
Feature selection	Adaptive Group LASSO	Non-parametric method to determine variables that provide incremental information for the cross-section of expected returns	Freyberger et al., 2020
Volatility forecasting	PCA, PLS	Forecasting models for achieving information integration improving the accuracy of volatility predictions	Poncela et al., 2011; Asgharian et al., 2013; Cepni et al., 2019; Li X. et al., 2022
Volatility forecasting	MIDAS-RV-PLS, MIDAS-RV-PCA	Forecast combination methods for information integration methods	Yan et al., 2022
Volatility forecasting	MIDAS-LASSO	Forecasting stock market volatility	Marsilli, 2014; Lu et al., 2020; Li R. et al., 2022
Path algorithm	Generalized LASSO	They investigate the generalized penalty problems using lasso penalties focused on computational aspects	Tibshirani and Taylor, 2011; Arnold and Tibshirani, 2016

### 3.2 Time series forecasting

Time series forecasting is important in any portfolio management task. AI algorithms have performed significantly better than traditional methods, especially in recent years with the introduction of deep learning methods. For example, one algorithm that could be considered traditional for this matter is Autoregressive Integrated Moving Average (ARIMA), which has already been outperformed by a large margin by LSTM (Siami-Namini et al., 2018). Other approaches used for forecasting that give state-of-the-art results are Gated Recurrent Unit (GRU) (Sadon et al., 2021), Seq2Seq (Mootha et al., 2020; Dash et al., 2023) combined with other deep learning approaches such as LSTM. Other deep learning-based forecasting methods have also prevailed in recent literature. One example is Generative Adversarial Networks combined with Gramian Angular Fields (Ghasemieh and Kashef, 2023). Convolutional Neural Networks (CNNs), traditionally employed for images and videos, find application in forecasting financial time series data (Kirisci and Cagcag Yolcu, 2022). They demonstrate superior performance compared to older, non-neural network-based methods. Deep learning-based methods for time series forecasting are prevalent in the literature and will continue to give state-of-the-art results in the foreseeable future.

### 3.3 Correlations, clustering, and network analysis

The multitude of market constituents and their interrelationships, coupled with specific structures, motivate the application of unsupervised machine learning techniques. These methods reveal underlying structures, simplify visualization, and introduce a form of ordering in the market space. While traditional market representation often relies on the risk-return relation for different asset classes, data-mining techniques, including complex information filtering, clustering, and graph theory supported by various machine learning methods, offer new approaches for diversification.

In the classical Mean-Variance approach to portfolio allocation, the optimal portfolio seeks to minimize the variance (σ_*P*_) while maintaining a specified portfolio return. Reliable empirical determination of a correlation matrix becomes challenging for financial markets when *T*<*N* or *T* approaches *N*, where the correlation matrix can become ill-conditioned and random to a large extent. As a result, the out-of-sample risk of an optimized portfolio exceeds the in-sample risk. Random Matrix Theory (RMT) (Mantegna and Stanley, 1999; Bouchaud and Potters, 2003; Kwapień and Drożdż, 2012) is a mathematical tool that allows us to analyze the dispersion of correlation matrix when applied to the financial market. The objective is to mitigate bias in future risk estimates (Potters et al., 2005) by simplifying the large correlation matrices (Bun et al., 2017). This is achieved by extracting the systematic part of a signal hidden in the correlation data. Giudici et al. (2022) extended the application of RMT, a minimum spanning tree (MST), and portfolio optimization techniques to ETF markets, assisted by robot advisors as a FinTech innovation.

Cluster analysis, a well-established unsupervised classification method, has proven valuable across various fields, including finance. It aids in visually positioning assets by revealing underlying similarities. From a different perspective, clustering simplifies markets by reducing dimensionality and complexity, facilitating portfolio optimization. Two main clustering algorithms are hierarchical and partitional, with hierarchical identifying nested clusters and partitional finding clusters simultaneously. However, a common challenge lies in the need for cluster validation and the lack of cluster stability (Tan et al., 2005).

The grouping methods used in the partitional clustering process are the classical K-means and the PAM (Partitioning Around Medoids) algorithm, which picks one stock from each cluster with the highest Sharpe ratio. Duarte and De Castro (2020) segment the assets into clusters of correlated assets, allocate resources for each cluster and then within each cluster by different partitional clustering algorithms (*K*-medoids PAM and Fuzzy clustering). Khedmati and Azin (2020) include *K*-means and *K*-medoids but also spectral and hierarchical clustering considering transaction costs for different data sets. Soleymani and Vasighi (2020) addresses a large portfolio dataset to find the most and least riskiest *K*-means clusters of stocks based on VaR and CVaR measures and working only on financial returns. In unsupervised learning, specifically within partitional clustering and using diverse time-series representations, a significant research direction involves applying fuzzy clustering to economic time series. For instance, D'Urso et al. (2013) and D'Urso et al. (2016) utilized a model-based approach with various fuzzy cluster variations and different distance metrics in financial markets. As an alternative to ultrametric spaces clustering methods, the Self-Organized Map (SOM) method was employed to cluster DJIA and NASDAQ100 portfolios, focusing on non-linear correlations between stocks (Zherebtsov and Kuperin, 2003). The authors concluded that the SOM method is more relevant and promising for clustering large, ill-structured databases requiring nonlinear processing.

The correlation matrix of financial time series can be used to arise hierarchical tree structures, taking the correlations ρ_*ij*_ as similarity measurement. The correlation-based clustering represented by network graphs allows for easy market visualization. On the standard methodology to build trees, for each pair *i, j* of assets, the distance *d*


(2) $$\begin{array}{l} d_{i,j}=\sqrt{2\left(1-\rho_{ij}\right)} \end{array}$$

is computed, where ρ_*ij*_ describes the correlation between log-return time-series. Having *d*_*i, j*_, we can compute MST or, equivalently, the Single Linkage Clustering Algorithms (SLCA) by using, for instance, Kruskal's algorithm. Such clustering analysis for portfolio optimization was explored by Tola et al. (2008). Marti et al. (2017) provides an in-depth overview of the state-of-the-art hierarchical clustering of financial time series. The hierarchical tree structure corresponds to diversification aspects in portfolio optimization models, where assets in the classic Markowitz portfolio are consistently located on the outer leaves of the tree (Onnela et al., 2002).

Network representation of complex financial markets offers a profound understanding of the underlying processes in the economic system, enhancing the information available to decision-makers. Analyzing stock market dynamics through network analysis can yield valuable insights and sound indicators for portfolio management (Battiston et al., 2016; Niu et al., 2021). The pioneering work on representing stocks as networks was published by Mantegna R (1999) where an MST was constructed based on the correlation among the stock prices for the DJIA and S&P 500 indices. Subsequent studies by the same group, summarized in Bonanno et al. (2004), extended MST applications to various stock markets and indices, exploring correlations with different time horizons. The concept of MST was further developed into dynamic MSTs in Onnela et al. (2002, 2003), revealing a scale-free property. During market crises, two network properties, normalized tree length and mean occupation layer from a central node (highest degree), decreased, indicating increased centralization. Additionally, stocks in optimal portfolios with minimal risks, as per the Markowitz model, tended to be in the network periphery, suggesting using network peripherality as an optimality indicator.

An alternative filtering approach for creating correlation-based Planar Maximally Filtered Graph (PMFG) was introduced in Tumminello et al. (2005), which produced graphs with a richer structure than MST, and further studied in Tumminello et al. (2006). The Directed Bubble Hierarchical Tree (DBHT) approach (Song et al., 2012) was explored in financial markets in Nicolo Musmeci and Tomaso (2014) and compared with MST and PMFG. Lower risk and better returns for more peripheral portfolios were demonstrated in Pozzi et al. (2013) using both MSTs and PMFG. This conclusion was reaffirmed more systematically in Peralta and Zareei (2016), introducing a ρ-based strategy for portfolio management that balances between the systematic (centrality) and individual properties of assets, confirming the performance of diversified portfolios with more considerable network distances. In Ren et al. (2017), peripheral portfolios perform better in stable periods with a drawdown in the investment horizon. In contrast, centrality-based portfolios are better for situations with a drawup in the selection horizon.

A particular case of applications is using network science and machine learning to build an HRP model (López de Prado, 2016). HRP models, part of the hierarchical approach, demonstrate robust out-of-sample properties without requiring a positive-definite return covariance matrix—a notable weakness in mean-variance-based portfolios. Different variants of this approach are proposed by Alipour et al. (2016); Raffinot (2017) improving the original HRP. Conceptually, HRP computes inverse-variance weights for groups of similar assets using an iterative process involving a correlation matrix. Additional steps include quasi-diagonalization, a rearrangement of the covariance matrix, and recursive bisection.

Recent stock market data analyses have employed Graph Neural Networks (GNN), enabling time-series data to be processed in a networked form within a deep learning pipeline. In Pacreau et al. (2021), portfolio management is formulated as a supervised learning problem using a multi-relational graph representation with sector, correlation, and supply-chain information. The authors employ various graph neural network architectures to solve this problem. A general framework for combinatorial optimization using graph neural networks is presented in Schuetz et al. (2022), which discusses its application to portfolio management. In works like Matsunaga et al. (2019); Chen Y. et al. (2018), graph neural networks are employed to incorporate companies' relationship data for stock price prediction, contributing to more informed decisions in portfolio management.

Additional applications for different purposes within this topic are described in Table 2.

**Table 2:** Applications of correlations, clustering, and network analysis.

**Application purpose**	**Method**	**Description**	**References**
Robust covariance matrix estimation	RMT	Analysis of the statistical structure of the empirical correlations and signal-noise separation based on the density of eigenvalues	Laloux et al., 2000; Frahm and Jaekel, 2005
Clustering-based stock selection	K-means, SOM, Fuzzy C-means	The clustering approach categorizes stocks listed in the Bombay Stock Exchange on specific investment criteria. The selected stocks from the clusters are used to construct a portfolio, aiming to minimize portfolio risk	Nanda et al., 2010
Clustering-based stock selection	K-means, PAM	A technique of portfolio construction based on establishing several portfolio positions are proposed, as well as choosing cluster representatives for the Warsaw Stock Exchange	Korzeniewski, 2018
Identifying market structures	Fuzzy PAM clustering, DTW distance	The proposed clustering method exploits dynamic time warping (DTW) distance to identify common time patterns for stocks composing the FTSE MIB index	D'Urso et al., 2021
Stock clustering	Cepstral-based fuzzy PAM clustering	Cepstral representation considers dynamic features in the clustering process. The approach efficiently clusters stocks based on the Sharpe ratio for each security	D'Urso et al., 2020
Industrial networks	Symbolic time series, hierarchical clustering, MST,	Symbolic representation reduce market dimensionality, and a hierarchical organization of DJIA companies is derived. The resulting clusters can be utilized to explore sector relationships and construct financial portfolios.	Brida and Risso, 2009
Stock network	Hierarchical clustering, MST	MST was established to represent the stock market by cross-correlations as a network	Mantegna R, 1999
Dependency modeling	Hierarchical clustering, MST	MST were constructed with links calculated using Pearson correlation for linear dependencies and mutual information for nonlinear dependencies. Utilizing the distance matrix and network measures from Onnela et al. (2002), the study revealed significant nonlinear correlations emerging during financial crises	Haluszczynski et al., 2017
Correlation regimes	Hierarchical clustering, MST	In a multi-asset futures portfolio, the framework establishes a macro-to-micro connection, classifying regimes at the macro level and characterizing individual markets based on their location within a network or cluster at the micro level	Papenbrock and Schwendner, 2015
Portfolio optimization	Networks, centrality measures,	Networks were created from the full cross-correlation and global-motion matrix. The study found that portfolios with more peripheral assets outperformed those with central assets. The beneficial role of eigenvalue decomposition of the system into market modes was demonstrated	Li Y. et al., 2019

### 3.4 Exploring the risk-and-return characteristics of asset classes

Asset allocation strategy involves forecasting risk-and-return characteristics for different asset classes or risk premiums. It includes determining the allocation percentages for each asset class in the portfolio. ML techniques offer a more efficient means for portfolio managers to handle expected values based on various forecasting models for risk and returns, considering for each case different risk measurements that distinguish downside from upside risk (Kuan et al., 2009; Harris et al., 2019; Liu and Wang, 2021; Mariani et al., 2022). The predictive models should be adapted depending on the target group of assets, considering traditional stocks, bonds or alternative investments (Fu et al., 2018). At this point, we mention the controversy in the literature about the evidence that there are real out-of-sample benefits to investors when relay on predictive models (Welch and Goyal, 2007; Johannes et al., 2014).

ML methods, with their high-dimensional nature, encompass diverse techniques, from traditional statistical learning methods like Gradient-Boosted Trees and Random Forest (RF) to the latest and popular algorithms such as Deep Learning (DL) or Deep Neural Networks (DNN). These methods use learning algorithms to identify the best-performing assets based on profitability and risk for a specific period. The goal of all of these methods is to approximate best the conditional expectation $\text{E}\left(r_{i,t+1}|F_{t}\right)$, where *r*_*i, t*+1_ is an asset's return over the risk-free, and $F_{t}$ is the actual and observable information set of market participants. Portfolio efficiency, gauged in profitability, is enhanced when assets are preselected based on return predictability, with the prominent application of ML techniques (Ballings et al., 2015; Kaczmarek and Perez, 2021). The most promising ML applications focus on finding predictive signals among the noise and capturing the alphas (Mirete-Ferrer et al., 2022). So, the goal is to achieve good indicators proven to detect successful companies in terms of stock-level signals combining different scores. In this way, the high amount of potentially good factors as signal makes ML effective for various reasons:

- ML is specially designed for forecasting purposes;
- It can cope with a large number of predictors and overcome the high dimensionality of the problem by combining many weak sources of information;
- Detection of nonlinear and complex relations and specially designed to mitigate overfitting;
- High sensitivity to low signal-to-noise ratios on the data;
- Avoiding crowded trades for highly correlated signals on different investors.

**Figure 4:**
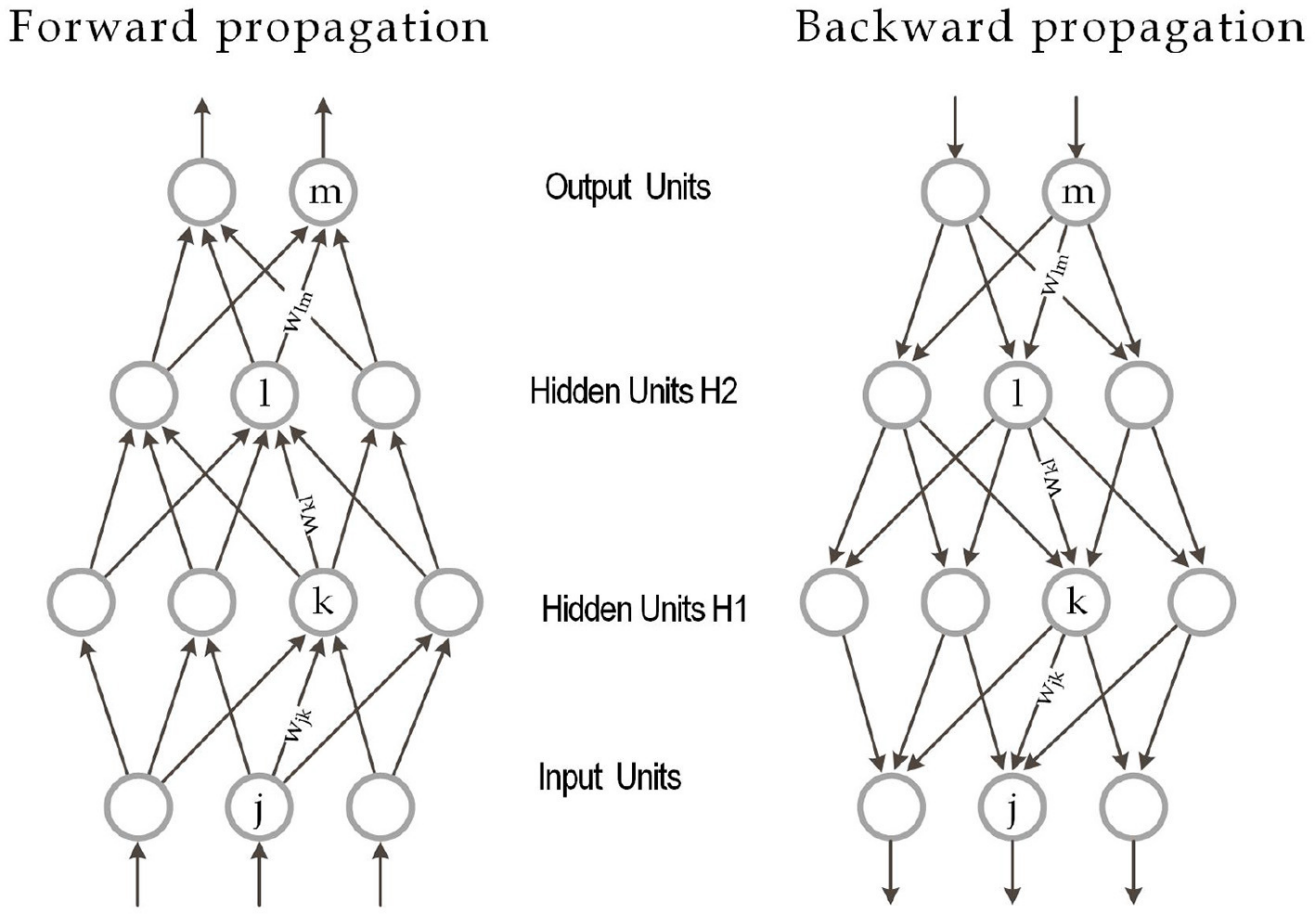
Multilayer neural network with forward and backpropagation and two hidden layers. Source: LeCun et al. (2015).

Deep Learning or deep neural networks algorithms refer to models represented in Figure 4 that consist of *L* layers or stages of nonlinear information. Each hidden layer takes the output from the previous layers and transforms it into an output as follows using the standard terminology stated in Lee et al. (2017); Hayou et al. (2019) for a fully connected random neural network of depth *L*, widths (_*N*_*l*_)1 ≤ *l* ≤ *L*_, weights $W_{ij}^{l}\overset{\text{iid}}{\sim }$ $N\left(0,\sigma_{b}^{2}\right)$. For some input *a*∈ℝ^*d*^, the propagation of this input through the network is given for an activation function ϕ:ℝ → ℝ:


$$\begin{array}{lll} y_{i}^{1}\left(a\right) & = & \overset{d}{\underset{j=1}{\sum }}W_{ij}^{1}a_{j}+B_{i}^{1},\\ y_{i}^{l}\left(a\right) & = & \overset{N_{l-1}}{\underset{j=1}{\sum }}W_{ij}^{l}\phi \left(y_{j}^{l-1}\left(a\right)\right)+B_{i}^{l},\;forl\geq 2. \end{array}$$

Indeed, an activation function ϕ decides whether a neuron should be activated and whether the input is important. Typically ϕ takes the rectified linear form Φ(*x*) = *ReLU*(*x*_*k*_) = *max*(*x*_*k*_, 0).

The more common activation functions besides *ReLU* are the following:


$$\begin{array}{l} ReLU:\phi (x)=max(0,x),\\ Sigmoid:\phi (x)=\frac{1}{1+e^{-x}},\\ Tanh:\phi (x)=\frac{1-e^{-2x}}{1+e^{-2x}},\\ LeakyReLU:\phi (x)=\{\begin{array}{cc} x & if\;x>0\\ 0.01x & otherwise \end{array}, \end{array}$$

and they have shown their utility in complex non-linear associations and, more generally, in selection problems.

These algorithms have demonstrated the potential to improve the implementation of different portfolio management strategies (Heaton et al., 2016; Grace, 2017) mapping data into the value of returns outperforming very different benchmark index, we can see an excellent example in Huang (2022) applying which is called Multitask Learning (MTL) for value extraction of hundreds of accounting terms in financial statement. The family of DL algorithms applied for portfolio construction is broad (Emerson et al., 2019; Ozbayoglu et al., 2020), and they are used in different stages of portfolio management.

We anticipate that Deep Learning, Reinforcement Learning, and Deep Reinforcement Learning applications in portfolio optimization will be specifically treated when we explain optimal portfolio construction techniques.

Random Forest (RF) is an ensemble ML algorithm introduced by Breiman (2001), employing a majority vote across individual decision tree learners. These non-metric models make no assumptions about data distribution and have fewer parameters to optimize compared to many other ML models. RF effectively handles complex signals like excess returns or risk premia, providing a good variance-bias trade-off and being reported as highly accurate learning algorithms. Additionally, RF models mitigate the impact of noise and changing relationships in past data between predictors and target variables, such as excess returns. Another popular approach is Gradient Boosting Trees (GBT), which builds trees sequentially, with each new tree aiming to correct the errors of the combined ensemble of the previous trees. GBT is typically applied to construct portfolios by leveraging their ability to predict asset returns and optimizing the portfolio based on those predictions. More examples of ML used for portfolio construction are displayed in Table 3.

**Table 3:** Picking attractive securities.

**Application purpose**	**Method/data**	**Performance criteria**	**References**
Measuring asset price premiums	Boosted RT, RF and NN	The higher gain of ML methods compared with leading regression-based strategies for return prediction is shown	Gu et al., 2020
Risk price estimation and dimensionality reduction	Bayesian approach	Building of a robust stochastic discount factor from a large set of stock characteristics	Kozak et al., 2020
Return estimation	RT	RTs were built to determine which firm characteristics out of 30 attributes are likely to drive future returns	Coqueret and Guida, 2018
Feature extraction	Restricted Boltzmann Machine	Proposes an encoder to extract features from stock prices and pass them to a feedforward NN	Takeuchi and Lee, 2013
Prediction of stock markets	RF	Method designed to predict price trends in the stock market	Kamble, 2017; Zhang et al., 2018
Cross-section prediction of exceed return	RF	Select stocks in S&P500 and STOXX600 with the highest monthly predictions	Kaczmarek and Perez, 2021
Benchmarking of ML techniques	RF, GBT, DL	Ensembles of different ML methods in the context of statistical arbitrage for S&P500	Krauss et al., 2017
Building ML signals for long-short strategies	GBT	Boosted Trees to more than 200 features clustered in six families, building an ML signal that outperforms the benchmarks for long-short strategies	Guida and Coqueret, 2018
Distinguish “good” stocks from “bad” stocks	LR, DNN, RF	Effectiveness of the stock selection strategy is validated in the Chinese stock market in both statistical and practical aspects where stacking outperforms other models	Fu et al., 2018

### 3.5 Enriching feature set by natural language processing

Natural Language Processing (NLP) coupled with Sentiment Analysis (SA) can assess the polarity of market signals in textual content from social media platforms—indicating whether sentiment is positive, negative, or neutral. Sentiment is used qualitatively and quantitatively to reflect opinions, attitudes, moods, or emotions toward securities, assets, companies, or the market. Some studies leverage existing sentiment indicators, while others calculate sentiment indexes. Data sources for sentiment analysis include news channels and social media, and approaches range from text representation methods to artificial intelligence classifiers (Mishev et al., 2020).

Microblogging services, like StockTwits, have become popular as investor-based social networks where users share investment opinions through microblogs. Evidence suggests that these opinions influence stock price movements, contributing to collective market sentiment. Additional sentiment analysis data sources include StockFluence sentiment data, aggregating opinions from various media channels, and Glassdoor, offering business outlook ratings from employee reviews. Twitter and Google are commonly used sentiment analysis data sources, with alternatives including sentiments extracted from Intrinio, Thompson Reuters, and Bloomberg news articles.

Another strand of literature covers the use of cutting-edge NLP approaches to process and distill the public mood, which may include polarity detection, micro text analysis, aspect extraction or sarcasm detection in different levels of granularity like entity level, sentence, document or context. In general, NLP-based sentiment analysis methods could be divided into two categories. First, NLP combined with traditional machine learning like SVM (Long et al., 2019), LightGBM (Wu et al., 2020), XGBoost and RF (Jourovski et al., 2020; Petropoulos and Siakoulis, 2021). Evidence supports that financial news or social media information can provide an additional advantage in predicting price or market turbulence trends. This approach often entails constructing numerous features before inputting them into the ML model. Alternatively, some studies explore DL techniques, which can automatically extract features from news or social media. For instance, a self-regulated generative adversarial network was proposed to enhance generalization and overcome stochasticity in predicting stock movements based on financial news and historical price data (Xu et al., 2022). Comparatively, a hybrid data analytics framework, integrating CNN and bidirectional LSTM, was created to predict stock trends by estimating the impact of news events and sentiment trends converging with historical financial data. Unlike other studies, LSTM was trained to automatically generate an asset allocation strategy using historical lagged data and public mood (Malandri et al., 2018). Similarly, in Xing et al. (2018), sentiment information is mapped to market views using a neural network design based on an ensemble of evolving clustering and LSTM. These views are integrated into modern portfolio theory through a Bayesian approach, and the portfolio's performance is analyzed for aspects like portfolio stability, sentiment time series computation, and profitability in simulations.

Financial sentiment analysis faces challenges due to specialized language and a lack of labeled data. The advent of ULMFit (Howard and Ruder, 2018) has facilitated effective transfer learning in NLP. For example, Feinberg (Bidirectional Encoder Representations from Transformers for financial data) is a pre-trained NLP model designed explicitly for sentiment analysis in financial text (Araci, 2019). Comparatively, Zhao et al. (2020) proposed a RoBERTa as a pre-trained model, which exploits different fine-tuning methods for sentiment analysis and critical entity detection in online financial texts. SEntFiN 1.0 is the most recent publicly available example of a human-annotated dataset of news headlines containing multiple entities (Sinha et al., 2022). The authors concluded that deep bidirectional pre-trained language models such as domain-specific BERT fine-tuned to SEntFiN outperform state-of-the-art learning schemes significantly.

Table 4 provides examples of papers focusing on sentiment signal generation for asset allocation.

**Table 4:** The use of sentiment signals for asset allocation.

**Application purpose**	**Method**	**Description**	**References**
Stock portfolio construction	RNN, LSTM, RF, MLP, StockFluence sentiment	The study explores whether public mood collected from social media and online news is correlated or predictive of portfolio returns by constructing five portfolios from 15 NYSE stocks	Malandri et al., 2018
Stock portfolio construction	Deep RL, market sentiment	Sentiment-aware deep deterministic policy gradients approach learns from historical stock price trends and market sentiments perceived from Google News and Twitter about 30 Dow Jones companies	Koratamaddi et al., 2021
Stock portfolio construction	Sentiment extraction, ML, weblogs	Ontology-guided and rule-based web information extraction based on domain expertise and linguistic knowledge with a focus on weblogs	Klein et al., 2011
Stock portfolio construction	Hierarchical Clustering, regime-switching, ML, market sentiment,	Regime-Based asset allocation models are proposed, where investors' mood swings interpret the regime. Then, the Black-Litterman asset allocation model is used to construct a portfolio	Zhang et al., 2020
Stock portfolio construction	Spectral Clustering, stochastic NN, beliefs,	Asymmetric investors' sentiments reflect market participants' beliefs about future cash flows. These sentiments, combined with investor results and previous sentiments, inform a dynamic investor sentiment-adjusted multi-period portfolio selection model	Wei et al., 2021
Stock market prediction	Kalman Filter, ML, microblogs, survey indices	The prediction model employs sentiment and attention indicators extracted from microblogs and survey indices (AAII and II, USMC and Sentix), the use of a Kalman Filter to merge microblog and survey sources, and then several ML methods	Oliveira et al., 2017
Stock selection	LR, LightGBM, analyst reports, reviews,	The study explores the impacts of analyst attitude and crowd sentiment on stock prices, indicating that crowd wisdom is more valuable than expert wisdom in shaping investment strategies.	Wu et al., 2020
Stock beta forecasting	LASSO, RF, XGBoost, news volume, stock sentiment,	Beta are estimated using sentiment-embedded machine learning models. Market-neutral long-short portfolios are then constructed, and feature importance is determined using the Shapley value.	Jourovski et al., 2020
Stock return prediction	Employee sentiment from Glassdoor	A proposed aggregate measure of employee sentiment, derived from millions of employee online reviews, is identified as a robust predictor of market returns	Symitsi and Stamolampros, 2021
Investment recommendation	Factor model, LR, StockTwist	To predict the quality of an investment opinion, various factors derived from author information, opinion content, and the characteristics of referenced stocks are employed	Tu et al., 2018
Feature extraction	Text representation methods, NLP, ML, SemEval-2017,	The study utilizes lexicon-based feature extraction methods, word and sentence encoders, and state-of-the-art NLP transformers. A deep-learning and transfer-learning-based sentiment analysis model, coupled with machine learning models, is applied for portfolio construction	Mishev et al., 2020

### 3.6 Examining the interrelation between ML and market efficiency

In classical economic theory, economists explore models with market frictions, where price competition may be dampened, leading to potential unemployment of resources. AI holds significant potential to enhance efficiency by reducing search frictions (Milgrom and Tadelis, 2018). AI aids in understanding market environments, identifying patterns that enhance customer experience, and improving forecasting to promote more efficient market operations. Indeed, determining evolving market conditions is mainly linked to capturing market inefficiencies to identify future performance. This is where the usefulness of the application of AI arises. Many studies demonstrate the superiority of AI over traditional ones. However, the question is how the massive use of information-based systems, for instance, supported by cloud services, can change the price discovery process. Unequal access to AI technology among financial actors may lead to smaller providers' limited participation, posing a concentration risk among more prominent players (Duan et al., 2019).

AI, particularly in High-Frequency Trading (HFT), generally introduces greater complexity to conventional algorithmic trading, notably in highly automated markets such as equities and FX. AI and HFT contribute to enhanced liquidity provision and enable the execution of large orders with low market impact. From a risk perspective, AI allows order flow management, reducing inefficiencies. HFT serves as a significant source of liquidity, so any disruption in their operation results in liquidity being pulled out, especially when AI techniques are widely deployed. At this point, we have to distinguish two significant impacts of the massive application of AI on the financial markets that result in two sides of the same coin. First, AI impacts information efficiency by reducing the marginal cost of information acquisition and processing for portfolio managers. Second, the question is how AI is going to replace human decision, as the machines process much more information faster, making the markets more efficient (Barbopoulos et al., 2021), but at the same time with a higher risk of market manipulation by using spoofing schemes as 2010 Flash Crash (U.S. Department of Justice Office of Public Affairs, 2015) being a source of non-financial risk.

In particular, analyzing the interrelation between AI and market conditions and how this relation changes sophisticated investors' behavior has just begun (Chen Y. et al., 2020). Regarding the first point, consider the quarterly annual reports for the Russell 3000 Index, which includes around 3000 of the largest U.S. companies, resulting in ~12,000 documents in a fiscal year. Managing such a vast amount of information is challenging for humans. An important distinction between humans and machines is that humans tend to pay more attention to large and value firms, whereas AI accesses information more uniformly (Barbopoulos et al., 2021). The studies on the interaction between information and potential impacts on market efficiency have to rely on accurate metrics. For instance, the Security and Exchange Commission's (SEC) Electronic Data Gathering and Retrieval (EDGAR) website allows researchers to measure with automatic algorithms how the stock market responds at the time of earning announcements. All internet search traffic of the EDGAR system is accessible to researchers, including the user's IP addresses and the user requesting the information. The impact of our trading decisions on the market and queries made through the SEC exchange requesting information from companies is observable. Table 5 provides the examples of paper, where the interrelation between AI and market efficiency was analyzed.

**Table 5:** Interrelation between AI and market efficiency.

**Application purpose**	**Method**	**Performance criteria**	**References**
Analysis of SEC reports and investor attention	SEC's EDGAR	The attention of sophisticated investors for the earning announcement impacting on portfolio performance is measured	Li R. et al., 2019
Analysis of endogenous information acquisition	SEC's EDGAR	A long-short portfolio based on different measures of information acquisition activity generates a monthly abnormal return of 80 basis points that is not reversed in the long-run	Li and Sun, 2022
Arbitrage trading strategy based on machine learning	LR, RF, Gradient Boosting Classifier	Volume-Weighted Average Prices (VWAP), ML models outperform the general market by far, which poses a clear challenge to the semi-strong form of market efficiency in futures markets	Waldow et al., 2021
ML algorithms to find profitable technical trading rules using past prices		Genetic algorithm, KNN, RF The out-of-sample profitability decreases through time, becoming the markets more efficient over time	Brogaard and Zareei, 2021
Analysis of cryptocurrency market efficiency	RNN applied to XBTEUR time series bitcoin market	Applying F-measures authors show that Bitcoin market is partially efficient	Hirano et al., 2018
Testing the weak-form efficient market	SVM and LR	Randomness of a sequence of rising/falling states of stock prices	Khoa and Huynh, 2021

### 3.7 Selection of particular assets using multiple criteria

Modern portfolio theory initially considered mean and variance as the sole criteria for portfolio selection. However, over the past 60 years, more sophisticated methodologies and techniques have been proposed, incorporating utility/desirability functions (Scott and Horvath, 1980; Neves et al., 2017), expectation-risk (Konno and Yamazaki, 1991; Speranza, 1993), requirements for higher moments of portfolio (Cvitanić et al., 2008), stochastic dominance (McNamara, 1998), etc. Furthermore, fundamental analysis (Greig, 1992; Mukherji et al., 1997) and technical analysis (Pinches, 1970; Austin, 1986; Chou et al., 1997; Yao et al., 1999), followed by factor analysis (Hui and Kwan, 1994) and attribute clustering (Huang and Jane, 2009), are sources for multi-criteria decision making (MCDM) (Colson, 1985).

One notable paper on multi-criteria portfolio selection is by Zopounidis (1999), where the author reviews decision-aid methods, their structure, and processes existing at that time. The paper also briefly explains how MCDM works in financial management. Comparatively, a significant analysis was presented by Aouni (2009), where the author linked portfolio optimization with multiattribute portfolio selection. In his further research (Aouni, 2010; Aouni et al., 2008), the author gave more examples of how goal programming can be used in portfolio selection. A comprehensive review of MCDM techniques was presented in the study Mardani et al. (2015), where a list of publications (more than 460) with different applications in many fields of science, engineering and management was provided. Among them are such techniques as AHP (Forman and Gass, 2001), PROMETHEE (Brans, 1982), ELECTRE (Roy, 1968), TOPSIS (Hwang and Yoon, 1981), ANP (Saaty, 1996), VIKOR (Yu, 1973), and hybrid MCDM (Shyur and Shih, 2006). However, they found only one publication, namely (Vetschera and Almeida, 2012), related to the portfolio selection problem. Later, Munhoz Arantes and Cesar Ribeiro Carpinetti (2019) published a review (with more than 110 papers cited) of how MCDM can be used for risk assessment. It has been emphasized that MCDM, coupled with the generalization of fuzzy sets, is gaining popularity among decision-makers and researchers. Specifically, Mohagheghi et al. (2019) suggested how MCDM should deal with uncertainty-related issues and which optimization techniques could be useful for project portfolio construction. Moreover, they reviewed real-world applications and case studies, excluding the financial portfolio selection problem. However, Liesiö et al. (2021) linked general project portfolios to financial portfolio selection and introduced so-called portfolio decision analysis techniques.

The abovementioned methods and techniques can help solve financial portfolio selection problems as alternatives to AI black-box techniques. Furthermore, Galankashi et al. (2020) provided a list of potentially attractive criteria and reviewed related works. Moreover, they applied fuzzy ANP and showed the entire decision-making process. Such a technique could be helpful in ANN's training phase.

Optimization-based approaches traditionally use technical and fundamental indicators to determine portfolio composition. Demand and supply of stock shares and market patterns are studied using technical analysis (Achelis, 2000). The basic indicators are based on information from each company's financial reports. Silva et al. (2015) applied evolutionary algorithms using several fundamental indicators [debt ratio, ROE (return on equity) and P/E ratio] together with technical indicators to generate optimal portfolios.

The repeatability of data patterns, the visual signals of indicators and oscillators, and the graphical representation of the evolution of assets are the sources for financial technical analysis (Turcaßs et al., 2016). Portfolio selection based on technical analysis implies the idea that prices move up (i.e., bullish), down (i.e., bearish), and sideways (i.e., trading) in a trend and that these trends ultimately influence the movement of financial assets.

Table 6 summarizes papers on MCDM and emphasizes the method, criteria used and application field.

**Table 6:** MCDM techniques used for portfolio selection.

**Method**	**Criteria used**	**Description**	**References**
PROMETHEE outranking method	Outranking-based approaches	A new formulation of the PROMETHEE V method was proposed, and several alternative methods based on the concepts of marginal and c-optimal portfolios were developed. The methods provide a good approximation of the PROMETHEE ranking of all portfolios, and their application requires only a small computational effort even for significant problems	Vetschera and Almeida, 2012
MCDM, DEA, Entropy, MABAC	Risk and return parameters	The performance of the funds is analyzed using Data Envelopment Analysis (DEA) to allow an initial selection of funds. Then, the Multi-Attribute Border Approximation Area Comparisons (MABAC) is applied, where the weights are calculated using the entropy to rank the funds according to risk and return	Biswas et al., 2019
Bayesian decision problem, multivariate skewness, utility function maximization	The mean, standard deviation and cubed-root of skewness	The skew-normal distribution were employed in a method for optimal portfolio selection using a Bayesian decision theoretical framework that addresses two significant shortcomings of the traditional Markowitz approach: the ability to handle higher moments and parameter uncertainty	Harvey et al., 2010
Multi-criteria utility functions, Multiple Criterion, Stochastic Programming	Portfolio return, dividends, growth in sales, social responsibility, liquidity, etc.	It summarizes multi-criteria portfolio selection approaches, answering the question of how to incorporate additional criteria beyond risk and return into the portfolio selection process	Steuer et al., 2008
ELECTRE, MCDM	Return on assets; Return on equity; Net profit margin; turnover; Cash liquidity; etc.	The ELECTRE Tri outranking method is used to provide a multi-criteria methodology to select stocks based on financial analysis	Xidonas et al., 2009
Multiple criteria, linear programming,	Mean-risk	The multi-criteria linear programming model for the portfolio choice problem is based on risk preferences. It enables standard multi-criteria techniques to analyze the portfolio choice problem. It is also demonstrated that the classical mean-risk methods used in linear programming models are consistent with the specific solutions applied to multi-criteria model	Ogryczak, 2000
Fuzzy analytic network process (FANP)	Profitability, growth, market, and risk	A fuzzy analytical network process (FANP) and specific criteria were developed to evaluate and select the stock portfolios	Galankashi et al., 2020

Table 7 emphasizes the purpose of the MCDM application. However, the method and criteria also are indicated.

**Table 7:** MCDM approaches used for particular purpose.

**Purpose of application**	**Method/data**	**Criteria used**	**Description**	**References**
Ranking of Stocks	MADM Methods, Financial Ratios, p-TOPSIS Method, p-VIKOR Method	Total Income (TI), Net Profit (NP), Net Worth (NW), Return on Net worth (RON), Stock Price (SP), Promoter Holding (PH), FII + DII Holding (FII), Operating Prof-it Margin (OPM), Net Profit Margin (NPM), Dividend Payout Ratio (DPR)	The model proposed in the study can provide more information on the overall performance of a particular share compared to other shares. The results obtained by the different methods clearly distinguish good companies from poorer ones, although the exact ranking varies slightly	Hwang and Yoon, 1981
Hybrid model for MCDM	TOPSIS, ANP, NGT, Multiple criteria analysis	Price/cost; On-time delivery; Product quality; Facility and technology; Responsiveness to customer needs; Professionalism of salesperson; Quality of relationship with vendor	The five-step hybrid process and the Analytical Network Process (ANP) method allow the relative weights of several assessment criteria to be determined using the Nominal Group Method (NGT)	Shyur and Shih, 2006
Decision making	Multi-Objective Programming (SMOP); Goal Programming (GP); ten stocks return rate of the Tunisian stock exchange	Return rate; the level of risk	To get the best solutions in decision-making situations a model of goal programming is formulated and a deterministic equivalent formulation of stochastic multi-objective optimization programs is considered	Aouni et al., 2008
Decision making	Analytic Hierarchy Process (AHP)	Theoretical background	Discuss why AHP is a standard methodology for a wide range of solutions and other applications and develop academic discussions regarding the effectiveness and applicability of AHP compared to competing methods by providing brief descriptions of successful applications of AHP	Forman and Gass, 2001
Asset allocation	Gray MCDM, gray-ANP, gray-DEMATEL, Shanghai Stock Exchange, China	Return, financial ratios, dividends, risk	This study uses a hybrid MCDM approach consisting of an integrated analytical network process (ANP) and a decision-making test and evaluation laboratory (DEMATEL) in a gray environment to select an optimal portfolio to provide decision-makers with both ranking and weighting information	Mills et al., 2020

In general, MCDMs are transparent decision-making tools compared to most AI techniques. However, it is heavily dependent on the decision-makers and pre-selected criteria.

## 4 Constructing the optimal portfolio

The most popular criteria in academic literature for constructing optimal portfolios are mean and variance of returns. However, such an approach leads to a quadratic optimization problem if constraints are no more complex than quadratic. Some authors suggested maximizing skewness (e.g., Konno and Suzuki, 1995) together with maximizing means and minimizing variance, which resulted in the optimization problem becoming much more complex as the utility function became cubic. Furthermore, some authors suggest using a utility function of even higher order (see Harvey et al., 2010 or Levy and Hanoch, 1970). The other approach is related to multi-criteria utility functions (see Steuer et al., 2008 or Ogryczak, 2000). Such types of utility functions lead to linear optimization problems. However, preparations require much more decision-maker involvement as criteria weighting is time-consuming. Moreover, the result is very subjective and may be biased as every decision maker may assign different weights (see Steuer et al., 2008, Galankashi et al., 2020). It is worth mentioning that many authors recommend including historical portfolio return, various security and systematic risk measures, dividends, liquidity, turnover, P/E, P/B, ROA, ROE, workforce, etc. Unsurprisingly, the factors mentioned above come from fundamental and technical analysis.

The following subsections discuss metaheuristics and ML optimization techniques used in portfolio optimization.

### 4.1 Metaheuristics for portfolio optimization

Portfolio construction, optimization, and management challenges have been extensively tackled using various metaheuristics, offering more flexibility in problem formulation than classical optimization approaches. Unlike the mean-variance model (Markowitz, 1959), these models can have a richer structure, and the optimization problem may be non-convex. While heuristic methods may compromise solution optimality, they often optimize more efficiently than classical methods. However, their effectiveness is problem-dependent, and formulating a more realistic model with numerous constraints, such as limiting the total number of assets or specifying bounds on each asset's quantity, can be relatively complex. An extensive survey of classical and heuristic optimization methods for portfolio optimization can be found in Mansini et al. (2014). Conversely, metaheuristic algorithms have a general problem-independent structure, although they may require tailoring to specific problems. Advances in parallel computing over the last decade have facilitated practical implementations of computationally intensive metaheuristic methods for large-scale complex problems. Metaheuristic algorithms can be categorized based on various aspects, including population-based or single-solution, naturally inspired, mimic evolution (evolutionary algorithm—EA), utilize swarm intelligence, involve global or local search, etc. These categories may overlap, and some algorithms are hybrid, incorporating techniques from multiple algorithm types. A broad introduction to various metaheuristic algorithms can be found in Talbi (2009). We will consider many of the metaheuristic algorithms, such as genetic algorithms (GA), evolutionary strategy (ES), differential evolution (DE), particle swarm optimization (PSO), ant colony optimization (ACO), artificial bee colony (ABC), simulated annealing (SA), quantum annealing (QA), and tabu search (TS). Some models have a single objective, like minimizing the variance, while others have multiple, like minimizing variance and maximizing return, which require an application of multi-objective evolutionary algorithms (MOEAs).

Table 8 summarizes some of the most critical applications of metaheuristic methods in portfolio optimization. For a comprehensive overview of MOEAs applied in portfolio management before 2012, the reader can refer to Metaxiotis and Liagkouras (2012). A recent survey on swarm intelligence techniques in portfolio optimization is available in Ertenlice and Kalayci (2018). Additionally, Doering et al. (2019) offers a broad survey covering various types of metaheuristic methods for both portfolio optimization and risk management.

**Table 8:** Applications of metaheuristic methods for portfolio optimization.

**Methods**	**Description, novelty and data**	**References**
GA, SA, TS	Application of cardinality constraints, examining GA and for the first time SA and TS, using data from 5-SMI which are later used in many other subsequent papers	Chang et al., 2000
TS	Including cardinality and bounding constraints on stocks from USA, UK, JP, DE and HK	Schaerf, 2002
SA	Incorporating cardinality, bounding, trading and turnover constraints on a dataset of 151 US stocks	Crama and Schyns, 2003
SA/ES	A hybrid model combining SA and ES examined with data from DAX 30 and FTSE 100	Kellerer and Maringer, 2003
ES	Multiobjective optimization using (1+1) ES on data from S&P 100 and some emerging markets	Fieldsend et al., 2004
GA, ES	MOEAs with cardinality constraints, buy-in thresholds and round lots on the HSI dataset of 31 assets	Streichert et al., 2004
GA, SA, TS	Multi-criteria model including individual preferences using multiattribute utility theory and S&P data	Ehrgott et al., 2004
GA	Replication of KOSPI 200 and TOPIX using a small number of stocks.	Orito et al., 2003; Oh et al., 2005
ACO, SA	Comparison of multiobjective optimization with ACO, SA and greedy search using data from 5-SMI	Armananzas and Lozano, 2005
E-MOEA	Envelope-based MOEA, a hybrid with parametric quadratic programming embedded among genetic operations tested on HSI, S&P 100 and Nikkei 225	Branke et al., 2009
GA	Besides cardinality constraints and bounding, incorporate transaction lots and market capitalization	Soleimani et al., 2009
DE	DE algorithm for Multiobjective Portfolio Optimization tested vs. NSGAII on Italian stock exchange	Krink and Paterlini, 2011
PSO	Cardinality constraints, bounding, transaction lots and market capitalization compared against GA	Golmakani and Fazel, 2011
PSO	Sharpe ratio as a fitness function and a comparison with GA using data from SSE 50	Zhu et al., 2011
ABC/FA	ABC algorithm hybridized with FA (ABC-FA) tested against NSGAII using 5-SMI data	Tuba and Bacanin, 2014
MODEwAwL	Learning-guided multi-objective evolutionary algorithm with external archive (MODEwAwL) compared with NSGAII, SPEA2, PESAII, PAES over I5 plus S&P 500 and Russell 2000	Lwin et al., 2014
MOEA/D	MOEA based on decomposition incorporating interval analysis examined using DJIA data	Solares et al., 2019
Multiple	Preselection procedures based on risk, return and correlation followed by optimization with NMOEA/D, MODE-SS, MODE-NDS, MOCLPSO, and NSGAII with data from Chinese stock exchange	Qu et al., 2017
TDMEA	3D encoding multiobjective EA (TDMEA) for large-scale problems tested on different model formulations using Nikkei 225, S&P 500, Russell 2000, and FTSE 100 data against NSGAII and SPEA2	Liagkouras, 2019
Reverse QA	Reverse QA with greedy search generated candidate solution is compared with forward QA and GA	Venturelli and Kondratyev, 2019
DE	Incorporating decision maker subjectivity in selection from solutions in a Pareto-front tested on DJIA	Fernandez et al., 2019
GA	Incorporating implicitly inferred decision-maker preferences and is tested with DJIA data	Fernandez et al., 2020

These five stock market indices (5-SMI), HSI, DAX 100, FTSE 100, S&P 100, and Nikkei 225, are used most often.

### 4.2 Deep learning, reinforcement learning, and deep reinforcement learning in portfolio optimization

DL concept has been used lately to manage portfolios in diverse conditions based on neural networks (Becker et al., 2019; Andersson and Oosterlee, 2021). Thus, numerous variants of DNN may function as independent evaluators to optimize the algorithm. The cryptocurrency market is often used in this type of research to evaluate the effectiveness of the DNN-based strategy compared to traditional portfolio management strategies (Sun et al., 2021). Some authors add fuzzy neural networks to the market forecasting when conditions change (Ghahtarani, 2021) dramatically. In other recent papers, a finite-time q-power RNN applied to solve the uncertain portfolio model is considered an improvement of classic NN (Ma and Yang, 2021).

Another solution to overcome the limitations of traditional and generic portfolio strategies considered in the recent literature is reinforcement learning (RL) using neural networks. This research direction argues for implementing RNN and conventional NN in reinforcement learning architecture to support investment decisions. The main element in this theory is the connection between agents and the environment (Sutton and Barto, 2018). As a fundamental component of the ML process, in RL theory, the agents are supported by NN to memorize and predict optimal decisions based on present information for an infinite number of actions and states (Wu et al., 2021). The environment then estimates the rewards from these actions to help agents learn for future decisions. This process can define specific models to gradually improve overall performance based on experiences gained with several trial and error steps.

In addition to this research direction, some authors claim that deep reinforcement learning (DRL) can be successfully used to capture the dependencies between the main features of some financial indicators, such as risk aversion, portfolio-specific characteristics and previous portfolio allocations (Benhamou et al., 2021b). At the same time, in deep consolidation learning, network composition and appropriate rewards significantly influence learning transactions in financial time series, using high-frequency data decomposed as input (Lee et al., 2021). A previous paper stipulated that portfolio management requires prior decisions as input to consider the effects of transaction costs, market impact or taxes, and this temporal dependence on the system's state involves reinforcement versions of standard recurrent learning algorithms (Moody et al., 1998). In another approach, DRL deals with low, high, and close prices through a designed depth convolution for these three characteristics. The classic methods cannot accurately estimate the critical time, so a three-dimensional warning gating network is used, giving greater importance to rising moments. Thus, deep-reinforcement learning tools obtain more substantial returns and improve profit indicators while reducing risk (Weng et al., 2020).

In other research, recurrent consolidation learning has successfully optimized portfolios. It memorizes up-to-date market conditions and constantly rebalances the portfolio's content based on classic performance indicators (Aboussalah and Lee, 2020). In some models, a compromise parameter is introduced to adjust the portfolio's optimism level, and learning algorithms evaluate market fluctuations and provide information to generate forecast hyperparameters. The main advantage of using these more complex methods is that the effectiveness and robustness of the portfolios obtained with their help significantly exceed the return and risk indicators obtained with the classical techniques (Min et al., 2021). Other methods study the relationships between financial instruments, which are considered to vary over time. These relationships are studied with the help of CNN, in which the market operator learns and applies an investment behavior that is constantly re-evaluated. Thus, the permanent reallocation of the assets from the portfolio is ensured to optimize the yield indicators (Soleymani and Paquet, 2021).

Recently, a new research direction has combined reinforcement learning and its applications with Python or similar programming languages coding to support understanding portfolio optimization mechanisms. These codes use dedicated open-source software as data processing media for programming (Graesser and Keng, 2019; Dixon et al., 2020). These research methods can integrate portfolio selection with portfolio optimization using multicriteria algorithms. The advanced programming languages with dynamic semantics allow every optimization step to be followed in detail, from the data entry to the extraction of the results (Sarmas et al., 2020). A significant advantage of using these methods is that free cloud-based platforms for programming effectively run the necessary programs (Rather, 2021). Thus, according to an increasing number of authors, Python or other programming languages can be used to build an efficient portfolio based on multiple optimization techniques to improve portfolio performance. Numerous results showed that the prediction models efficiently obtained high accuracy and enhanced yields (Ta et al., 2020).

As seen from the above, regardless of the method proposed for research, most papers cited conclude that optimizing portfolios based on DL, RL, or DRL have significantly better results than traditional algorithms. The generally accepted assertion is that these modern tools are superior to even the most advanced methods based on classical instruments. Moreover, using advanced programming languages, such as Python, supported by powerful open-source software and free cloud-based platforms, leads to superior results in optimizing portfolios, increasing returns and reducing risk.

## 5 Portfolio execution

This section focuses on executing portfolio orders and aligning them with investor objectives while considering market impact and asset price dynamics. Execution orders are a crucial element in portfolio management, closely linked to preceding portfolio rebalancing decisions. This integrated approach involves two interconnected facets. The application of established machine learning techniques, such as supervised and unsupervised learning (e.g., clustering, LASSO, Bayesian networks, and SVMs), becomes increasingly relevant. These techniques apply to portfolio execution, managing multiple variables such as order size, trade-quote relationships, order book imbalances, and spreads.

### 5.1 Rebalancing technique

Rebalancing, a crucial aspect of portfolio management, entails adjusting asset weights to maintain desired allocations or manage risk levels. This involves diverse strategies, from widely adopted to less conventional approaches. This comprehensive review explores these strategies, analyzing their characteristics, advantages, and limitations. The term “rebalancing” emphasizes adjusting asset weights to realign with chosen allocations or risk levels over time, without the necessity of adhering to a 50/50 stock and bond split (Tokat and Wicas, 2007; Kitces, 2015; Hong, 2021). Whether targeting a 50/50, 70/30, or 40/60 allocation, portfolio rebalancing involves reshuffling assets to achieve a predefined composition (Chen J. et al., 2020). Recognizing the diversity of rebalancing methods is crucial; some strategies are well-documented for their simplicity and effectiveness, while others, though less familiar, offer innovative perspectives. The table below summarizes and categorizes these types.

Assessing risk and return within a target asset allocation often relies on a rebalancing strategy. This approach considers the frequency of portfolio reviews, acknowledging it as a factor influencing whether the portfolio's actual performance aligns with its intended asset allocation. The core objective of rebalancing is to manage risk concerning the target asset allocation, prioritizing risk management over solely maximizing returns. Investors typically choose a rebalancing strategy based on their risk tolerance about expected returns, factoring in rebalancing costs (Zilbering et al., 2015). There isn't a universally optimal rebalancing frequency or threshold, as risk-adjusted returns tend to exhibit minimal differences among various rebalancing strategies (Tsai, 2001; Eakins and Stansell, 2007; Zilbering et al., 2015; Gruszka and Szwabiński, 2020).

Acknowledging the diversity of rebalancing methods is crucial; some strategies are well-documented in the literature for their simplicity and effectiveness, while others, though less familiar, offer innovative perspectives. Table 9 summarizes and categorizes these types.

**Table 9:** Summary and categorization of rebalancing strategies.

**Strategy type**	**Description**	**Nature**	**Focus**	**References**
Buy-and-hold strategies	Maintaining initial allocation over the investment horizon, relying on market recovery	Static	Strategic	Perold and Sharpe, 1988; Feldman et al., 2015; Hilliard and Hilliard, 2015
Calendar-time Rebalancing	Rebalancing at fixed time intervals, aiming to maintain desired allocation	Static	Strategic	Dayanandan and Lam, 2015; Lee et al., 2017; Chen J. et al., 2020; Lim et al., 2022
Risk-Parity Strategies	Allocating based on risk contributions for balanced risk exposure across asset classes	Dynamic	Tactical	Chaves et al., 2011; Roncalli, 2013; Costa and Kwon, 2019
Portfolio-insurance-based strategies	Protecting the portfolio from losses during downturns include constant-proportion Portfolio Insurance (CPPI), Option-Based Portfolio Insurance (OBPI)	Dynamic	Tactical	Zhu and Kavee, 1988; Bertrand and Prigent, 2005; Hong, 2021
Constant Mix Rebalancing	Maintaining a fixed allocation, rebalancing when deviations occur, buying low and selling high	Dynamic	Tactical	Jones and Stine, 2005; Cesari, 2011; Bertrand and Prigent, 2022
Threshold Strategy	Rebalancing when allocations exceed specified thresholds	Dynamic	Tactical	Zilbering et al., 2015; Lim et al., 2022
Time-Threshold Strategy	Combining time-based intervals and threshold triggers for rebalancing	Dynamic	Tactical	Daryanani, 2008; Dayanandan and Lam, 2015
Tactical Asset Allocation (TAA)	Making dynamic adjustments based on the market outlook for short-term opportunities and risk mitigation	Dynamic	Tactical	Weigel, 1991; Lee, 2000; Kanuri et al., 2021

Rebalancing strategies have been a subject of interest in various studies and research efforts. Perold and Sharpe (1988) categorized these strategies into four distinct approaches: buy-and-hold, constant mix, constant-proportion portfolio insurance (CPPI), and option-based portfolio insurance (OBPI). CPPI gained widespread adoption due to its ability to align asset allocation decisions with predetermined minimum dollar values (Zandieh and Mohaddesi, 2019). Moving ahead, Daryanani (2008); Zilbering et al. (2015); Dayanandan and Lam (2015) emphasized fundamental strategies, which included: (i) time rebalancing, (ii) threshold rebalancing, and (iii) a time-threshold rebalancing. These studies collectively underscored the significance of maintaining simplicity and consistency in portfolio maintenance.

Recently, Chen J. et al. (2020) introduced a structured framework categorizing rebalancing strategies into three primary approaches: calendar rebalancing, constant-mix strategy with bands, and CPPI. Calendar rebalancing involves periodic adjustments at fixed intervals, such as monthly or quarterly, regardless of market conditions. In contrast, corridor strategies set thresholds or bands around target allocations, prompting rebalancing when assets deviate beyond these bounds. Additionally, more recent research by Lim et al. (2022) has expanded the discussion by considering transaction costs, identifying two distinct approaches: complete portfolio rebalancing and gradual portfolio rebalancing. Complete portfolio rebalancing targets swift asset reallocation within a single trading day, while gradual rebalancing spreads adjustments across multiple trading days to minimize costs.

Customizing rebalancing strategies to consider specific factors like time constraints, transaction costs, and allowable deviations is vital. One adaptable method is threshold rebalancing, using range-based mechanisms to reallocate assets when they exceed predefined thresholds swiftly. Combining periodic and threshold strategies results in a hybrid approach that selectively rebalances portfolios when predetermined thresholds are breached. In the context of range rebalancing applied to portfolio benchmarks, asset classes are returned to their target allocations when they fall outside rebalancing bands. This approach underscores the importance of regular portfolio review and rebalancing only when asset allocations surpass a predetermined minimum rebalancing threshold. Moreover, rebalancing can also respond to tactical tail-risk models, highlighting the need for flexible portfolio management approaches (Packham et al., 2017).

### 5.2 Dynamic portfolio rebalancing with the help of AI/ML

The term “dynamic” denotes a strategy's ability to adapt swiftly to changing market conditions, asset performance, or specific triggers, diverging from predetermined time intervals (Perold and Sharpe, 1988, 1995; Bansal et al., 2004). Dynamic rebalancing, as articulated by Ilmanen and Maloney (2015), is an active investment approach where investors adjust their portfolios not confined to fixed schedules or specific percentage deviations. Instead, they realign portfolios with desired risk levels based on real-time market conditions. Diverging from traditional rebalancing methods, dynamic rebalancing is flexible and responsive, utilizing monthly market trends to dictate when and how much to rebalance while emphasizing exceptional signals in different asset classes. This approach aims to optimize investment performance while effectively managing risk (Gaivoronski et al., 2005).

There are both established and emerging techniques in dynamic portfolio rebalancing. Well-established methods include CPPI, OBPI, time-Threshold Strategy, and TAA, which have demonstrated their ability to enhance portfolio performance regarding risk-adjusted returns over many years.

The advent of AI/ML tools has ushered in a new era of dynamic portfolio rebalancing strategies. These emerging techniques harness the power of artificial intelligence and machine learning, offering innovative solutions. They encompass dynamic portfolio rebalancing through reinforcement learning (RL), utilizing its algorithms to maximize portfolio returns, and applying lag-optimized trading indicators in conjunction with genetic algorithms. To provide a practical glimpse into dynamic rebalancing, Jiang et al. (2020) developed a framework that integrates machine learning models into portfolio rebalancing, focusing on risk-aversion adjustment. This approach outperformed benchmarks in terms of returns and risk. Lim et al. (2022) employed an RL agent, introducing four distinct combinations of portfolio adjustments and price prediction models: (1) complete portfolio balancing without the Long Short-Term Memory (LSTM) prediction model, (2) complete portfolio balancing with the LSTM prediction model, (3) gradual portfolio balancing without the LSTM prediction model, and (4) gradual portfolio balancing with the LSTM prediction. Therefore, portfolio rebalancing utilizing the Recurrent RL (RRL) method and an adjusted objective function considering transaction costs and market risk aligns to develop efficient learning algorithms in RL, as discussed by Szepesvári (2010). Furthermore, RL has diverse applications in finance, including optimizing insurance pricing, bank marketing, portfolio management, and trading, as highlighted by Lim et al. (2022). Additionally, Jiang et al. (2020) integrated machine learning models into a portfolio rebalancing framework, adapting risk levels based on market trend predictions and consistently surpassing benchmark performance.

Nonetheless, it's essential to note that the effectiveness of these strategies may vary depending on factors such as portfolio size, investment objectives, and prevailing market conditions. Among the most recent and relevant studies Yeo et al. (2023) introduced two rule-based dynamic portfolio rebalancing algorithms: Tactical Buy and Hold (TBH), utilizing the forecasted Moving Average Convergence Divergence Histogram (fMACDH) indicator and risk differences and Rule-Based Business Cycle (RBBC), leveraging market sector performance variations across business cycles.

## 6 Portfolio evaluation: measurement, attribution, and appraisal techniques

### 6.1 Measurement

While the early literature on portfolio performance evaluation dates back to the 1960s, recent decades have witnessed a proliferation of novel methodologies, techniques, and empirical research in this field. These metrics effectively gauge the returns generated by a managed portfolio compared to the performance of a designated benchmark portfolio over a specific assessment period. Consequently, the benchmark portfolio must serve as a viable investment alternative for the managed portfolio under scrutiny (Brinson et al., 1995; Aragon and Ferson, 2006). However, Grinblatt and Titman (1989) introduces a comprehensive model designed to offer a nuanced perspective on diverse aspects of portfolio performance measurement. Within this model, a critical examination of various performance metrics unfolds, shedding light on their multiple criticisms. These criticisms encompass challenges like selecting an appropriate benchmark portfolio, the potential overestimation of risk due to market-timing skills, and the paradox of informed investors not realizing positive risk-adjusted returns due to growing risk aversion. Notably, the article contends that these significant issues should not be considered insurmountable obstacles in performance evaluation.

Portfolio performance evaluation assesses how a managed portfolio has performed compared to a specified benchmark. The methods for performance evaluation can be broadly categorized into conventional and risk-adjusted methods. Benchmark comparison and style comparison are prominent traditional methods, while risk-adjusted methods, including the Sharpe ratio, Treynor ratio, Jensen's alpha, Modigliani and Modigliani, and Treynor Squared, adjust returns to consider variations in risk levels between the managed portfolio and the benchmark portfolio. Preference is often given to risk-adjusted methods over conventional ones (Modigliani and Modigliani, 1997; Samarakoon and Hasan, 2013, 2022; Tamplin, 2023).

The conventional method, encompassing benchmark and style comparisons, assesses investment portfolio performance against a broader market index. Outperformance is determined if the portfolio's return exceeds that of the benchmark index over the same periods (Brinson et al., 1991; Samarakoon and Hasan, 2022). However, Aragon and Ferson (2006); Dor and Jagannathan (2002) have emphasized limitations, pointing out that this method may not consider variations in risk levels between the two portfolios. The portfolio might seem superior due to higher risk, leading to potential validity issues in a straightforward comparison.

Risk-adjusted approaches commonly alter returns to account for variations in risk levels between the managed and benchmark portfolios. As previously mentioned, we distinct, in the following, the most known and used approaches (see Table 10).

**Table 10:** Summary of risk-adjusted performance measures.

**Application purpose**	**Method/data**	**Description**	**Paper**
Portfolio performance	Sharpe ratio	Sharpe analyzed mutual fund performance, introducing the Sharpe ratio as a fundamental risk-adjusted performance metric. The Sharpe ratio continues to be crucial for assessing portfolio performance and plays a significant role in empirical asset pricing	Ferruz and Vicente, 2005; Aragon and Ferson, 2006; Samarakoon and Hasan, 2013
Portfolio performance	Treynor ratio	Treynor employed the market risk represented by beta stock. The Treynor ratio emphasizes systematic risk, ranks portfolio performance, and assesses diversification adequacy. Grounded in the Security Market Line, it compares the expected total return of a security or portfolio with that of a market portfolio	Hübner, 2005; Beer et al., 2011; Verma and Hirpara, 2016; Robiyanto, 2018
Portfolio performance	Jensen's Alpha ratio	Alpha are considered one of the most widely used traditional measures of investment performance. Thus, Jensen's Alpha is unique and considers systematic risks. A tool used for assessing the relative performance of a portfolio in comparison to benchmarks	Jensen, 1968; Hübner, 2005; Samarakoon and Hasan, 2013
Portfolio performance	Modigliani and Modigliani ratio	This metric presents an alternative risk measure, utilizing return volatility within the CAPM framework. The adjusted portfolio is constructed and managed as a blend of the managed portfolio and a risk-free asset, ensuring it matches the total risk of the market portfolio	Modigliani and Modigliani, 1997; Samarakoon and Hasan, 2022.
Portfolio performance	Multibeta Models	Multibeta models emerge when investors ideally hold combinations of a mean–variance efficient portfolio plus hedge portfolios for the other relevant risks. Most asset-pricing models describe the cross-section of expected returns regarding risk factor exposures, or betas	Sharpe, 1977; Velu and Zhou, 1999; Balduzzi and Robotti, 2008.
Portfolio performance	Weight-Based Performance Measures	A manager with investment ability raises the fund's exposure to securities or asset class before it performs well, or who expects and avoids losers	Aragon and Ferson, 2006; Ferson, 2013.
Appraisal ratio	Treynor–Black Appraisal Ratio	In this scenario, Treynor and Black (1973) calculate the mean–variance optimum portfolio and show that the optimal deviations from the benchmark holdings for each security are affected by the “Appraisal Ratio”. The ideal portfolio should include covered securities and index funds	Treynor and Black, 1973; Kahneman and Tversky, 2013.
Market Timing	Merton-Henriksson Market Timing Measure	This model allows the manager to monitor a private signal about the market's future performance. It changes the portfolio's market exposure or beta at the start of the period. However, the resulting convexity can be described with put or call options	Henriksson and Merton, 1981; Henriksson, 1984; Ferson, 2013.

### 6.2 Attribution

The field of performance attribution provides valuable insights for delineating investment responsibilities and measuring the contributions of various activities within the investment management process. Performance attribution seeks to clarify portfolio performance relative to a benchmark and pinpoint the origins of excess returns attributable to active decisions made by the portfolio manager. Bacon (2019) traces its evolution, beginning with Fama decomposition in the 1970s and progressing through subsequent developments, including multiperiod and multicurrency attribution in the 1990s, to contemporary models focused on fixed-income and risk-adjusted attribution. Bacon's comprehensive examination encompasses various attribution methods, such as returns-based, holdings-based, and transaction-based approaches, alongside considerations of money-weighted attribution and advancements related to notional funds.

In this historical context, Brinson and Fachler (1985) along with Brinson et al. (1995) established the basis for equity performance attribution, distinguishing excess returns into asset allocation, security selection, and interaction elements, ensuring they collectively constitute the active return. Extending this framework, Ankrim and Hensel (1994) incorporated currency management effects, introducing terms for currency forward premiums and surprise effects. These decomposition models remain relevant, as exemplified in Chen F. et al. (2018) examination of managerial skills.

Moreover, Fisher and DAlessandro (2019) introduced a novel risk-adjusted performance attribution analysis that integrates risk measures with Brinson models. This approach decomposes excess portfolio return into risk, allocation, and net selection components, ensuring additivity and consistency with financial theory. The risk adjustment can utilize either the traditional beta for Jensen's alpha calculation or Fama's beta, incorporating unsystematic risk while relying on relative standard deviations for risk adjustment in the Brinson attribution analysis.

### 6.3 Appraisal techniques

AI-based portfolio appraisal techniques offer several advantages, including optimizing trade timing, project performance evaluation, cognitive bias reduction, and improved decision-making. Some specific methods encompass Equal Weighted Portfolio (EWP), which assigns equal weight to each stock in a portfolio, irrespective of its company size, to reduce concentration risk and increase diversification (Malladi and Fabozzi, 2017; Lee, 2020). Inverse Volatility Portfolio (IVP) is another technique that helps in risk-adjusted allocations, performance evaluation using extensive data analysis, real-time detection and mitigation of decision-making biases, and the analysis of fundamental and alternative datasets to identify fresh investment prospects (Hallerbach, 2015; Rao, 2021).

Moreover, Thethi et al. (2021) recommends using LSTM for stock market prediction, surpassing traditional methods in performance. Shukla et al. (2022) focuses on improving financial portfolios through machine learning, considering the user's risk profile and employing ML for stock selection and capital allocation. Boudabsa and Filipović (2022) introduces a simulation approach for dynamic portfolio valuation and risk management, leveraging machine learning with kernels, demonstrating favorable outcomes in extensive dimensions. Kaczmarek and Perez (2021) illustrates that portfolio optimization techniques, such as Markowitz mean-variance and HRP optimizers, can enhance the risk-adjusted return of portfolios constructed with stocks preselected using ML.

In the context of real estate portfolio appraisal, Viriato (2019); Kok et al. (2017) exemplifies how Automated Valuation Models (AVM) have garnered substantial technological investment. These models can swiftly appraise many assets, streamline processes like property selection, expand investor access, facilitate efficient tax assessments, and improve understanding of value determinants. With the ongoing advancement of ML techniques, as demonstrated by Conway (2018), investors can leverage more precise valuation algorithms, effectively navigating a broad spectrum of opportunities.

## 7 *Post-hoc* explanations using XAI to build trust for portfolio management

### 7.1 Transparency and explainable AI on financial markets

The transparency and clarity of models using artificial intelligence are hotly debated. It is crucial for financial institutions, banks, governments or any other body that uses AI to trust the tools provided by researchers (Dwivedi et al., 2021; Ng et al., 2021). The AI tools and methods are not yet widely known to the general public, leading to a lack of confidence in the results obtained. Thus, it is trying to reach the concept of Responsible Artificial Intelligence as a methodology for the widespread implementation of AI methods in real life with correctness, explainability and responsibility (Arrieta et al., 2020). Deep reinforcement learning has recently been introduced to support socially responsible investments and portfolio optimization to achieve superior financial performance and a significant social impact (Vo et al., 2019). It is increasingly clear that the development of research in this area is closely linked to the capacity to ensure the transparency and explainability of the proposed models.

The increasing application of ML techniques to build portfolios and the concern in parallel on the ethical dimension of AI increases the interest in understanding how the different features interact and impact the model portfolio performance. At this point, XAI ensures the acceptance and adoption of AI-driven services and products. Still, it is only one of the four categories of trustworthiness technologies for machine learning, namely Fairness, Explainability, Auditability and Safety (FAES) (Toreini et al., 2020). At this stage, the starting point is the map of social sciences concepts such as ability, benevolence, integrity or predictability and linking these with AI framework showing their behavior in understandable terms for humans (Doshi-Velez and Kim, 2017). In this way, XAI helps compare different models and create rules for decision-making in which the underlying model can be explained to the users.

The concept of Shapley value introduced in cooperative game theory tells us how much each feature contributes to a specific result of the ML model (Lundberg and Lee, 2017; Lundberg et al., 2018). SHapley Additive exPlanation (SHAP) methodology allows us to quantify a model in an agnostic way, and we can see two approaches to address the interpretability issue of ML (Joseph, 2020): variable attributions via the decomposition of individual predictions (local attribution) and importance scores for the model as a whole (global attribution). Shapley values are the contributions of each feature to the overall crash logit probability. From the methodological perspective, the interpretation approaches for deep learning also have two main categories: surrogate model and feature importance extraction. Surrogate models use an explainable model, such as a decision tree, rule sets, linear models or generalized additive models, to proxy the neural network to be interpreted (Cong et al., 2020). Following are some examples of the application of the SHAP methodology. For instance, Jaeger et al. (2021) regress the Calmar ratio spread of HRP vs. Equal Risk Contribution (ERC) against statistical bootstrapped features applying Shapley framework showing insightful explanations. Schwendner et al. (2021) present a conceptual framework named Adaptive Seriational Risk Parity (ASRP) to extend HRP as an asset allocation heuristic using the SHAP framework to explain the resulting performance with features of synthetic market data. Also, referring to synthetic data, Papenbrock et al. (2021) evaluates three competing machine learning methods to regress the portfolio risk spread between both allocation methods against statistical features of the synthetic correlation matrices and then discusses the local and global feature importance using the SHAP framework. Benhamou et al. (2021a) apply Shapley values to provide a global understanding and local explanations of a proposed gradient boosting decision tree (GBDT) to plan regime changes of S&P 500 from a set of 150 technical, fundamental and macroeconomic features.

At this stage, it is interesting to bring here the debate of the dichotomy between the accurate Black Box and the not-so-accurate transparent model where Rudin and Radin (2019) referred to as complexity bias. We tend to find the complex more appealing than the simple. The belief that accuracy must be sacrificed for interpretability is inaccurate, and it explains the problems that have resulted from the use of black box models for high-stakes decisions throughout society, mainly in the finance domain. Going further, trusting a black box model means trusting the model's equations and the entire database from which it was built. In this way, for instance, Philps et al. (2021) proposes a symbolic artificial intelligence (SAI) for stock selection, a form of satisfying, provides an alternative to factor investing and overcomes the interpretability issues of many machine learning (ML) approaches by applying learns simple, interpretable investment rules using the non-linear power of a simple ML approach.

XAI, as an extension of ML techniques, is directly applicable to another use-case on finance linked to the automated management of the asset portfolio allocation. This leads us to the concept of Robo-Advisors (RAs) who offer automated online portfolios, which are currently one of the significant parts of the Fintech Revolution and likely one of the most disruptive trends in wealth and asset management nowadays (Beketov et al., 2018). The takeover of the robots-assets under management in the RA segment is projected to reach US$1,427,650m in 2021 with an annual growth rate 2021 of 34.8% and foreseen 18.78% yearly growth in 2021–2025, resulting in a projected total amount of US$2,842,101 m by 2025 Statista (2021). Two parts of the process operated by RA are crucial: (1) client profiling and (2) asset allocation. There is an abundance of research that demonstrates the interest in the subject and others that shows how RA improves portfolio performance (D'Acunto and Rossi, 2020; Hong et al., 2020; Rossi and Utkus, 2020; Bianchi and Briere, 2021a,b). The counterpart of this new trend is the increased opacity, missing accountability, transparency and financial inclusion. At his point, it becomes essential to design and implement trusted AI-based systems (Toreini et al., 2020) and again, XAI emerges as one of the most critical contributions from the ML landscape.

XAI has developed processes that explain already trained neural networks based on generating synthetic data in another research direction. It is a complex discussion about which XAI method gives the best results and whether the explanations can be reliable (Arras et al., 2022). A comprehensive approach to generating synthetic data uses a Generative Adversarial Network (GAN). Some authors have explored the possibilities of overcoming the difficulties of establishing the correct set of hyperparameters in the case of GAN by using reinforcement learning and Bayesian optimizations. They combine the Multi-Model-based Hybrid Prediction Algorithm with the GAN-based Hybrid Prediction Algorithm. Further, they obtained an improved model named Multi-Model Generative Adversarial Network Hybrid Prediction Algorithm for stock market prices prediction (Polamuri et al., 2021). The learning process approach based on a predictive probabilistic neural network corresponds to a different way of using training in the Conditional Generative Adversarial Networks (cGAN) as a predictive model in portfolio optimization, stock market prediction and trade execution (Zhang et al., 2019; Lee and Seok, 2021).

Summarizing XAI subject, there is no doubt how ML is becoming mainstream for investment management (Table 11), but the debate that has only just begun is how most ML approaches suffer from black-box problem, which is part of the agenda in increasing finance group discussions arising XAI as a key tool for Customers, Regulators, Practitioners and Risk Managers.

**Table 11:** Transparency and explainability.

**Application purpose**	**Method/data**	**Description**	**References**
Transparency and XAI	Responsible AI, Deep Responsible Investment Portfolio (DRIP)	The transparency and explainability of models using AI are crucial for any organization that uses AI. AI tools often lack widespread public awareness, contributing to a lack of confidence in their results. Encouraging professionals from various fields to understand the benefits of AI in their activities is crucial, overcoming initial reluctance due to perceived lack of clarity	Vo et al., 2019; Arrieta et al., 2020; Dwivedi et al., 2021; Ng et al., 2021
Identification of how ML impact trust frameworks are drawn	ABI (Ability, Benevolence, Integrity) principles	Authors identify how trust can be enhanced in the various stages of an AI-based system's life-cycle, specifically the design, development and deployment stages (AI chain of trust)	Toreini et al., 2020
Evaluation of interpretability	Taxonomy of Interpretability Evaluation	Authors hypothesize factors that may be the latent dimensions of interpretability (global vs. local; Area, Severity of Incompleteness; Time constraints; Nature of User Expertise)	Doshi-Velez and Kim, 2017
Understanding why a model makes a specific prediction	SHapley Additive exPlanation (SHAP)	Presentation of several different estimation methods for SHAP values, along with proofs and experiments showing what values are desirable	Lundberg and Lee, 2017; Lundberg et al., 2018
Insights qualifying generic statistical learning processes	Shapley-Taylor decomposition for a generic inference framework	Estimation of heterogeneous treatment effects in simulated and real-world randomized experiments	Joseph, 2020
Portfolio construction	Reinforcement-learning-based portfolio management	Authors propose a polynomial-feature-sensitivity (and textual-factor) analysis to project the model onto linear regression (and natural language) space for greater transparency and interpretation	Cong et al., 2020
Benchmark rule-based investment strategies	Calmar ratio spread between HRP and ERC	Authors regress the Calmar ratio spread of portfolio allocation backtests against statistical features of bootstrapped futures return datasets using XGBoost and apply the SHAP framework to discuss the local and global feature importance	Jaeger et al., 2021
Asset allocation heuristic	ASRP	Benchmarking of the standard HRP with other static and adaptive tree-based methods in backtests, as well as ASRP methods by SHAP framework	Schwendner et al., 2021
Construction of robust investment portfolios	Evolutionary algorithms to simulate synthetic correlation matrices	An explainable machine learning program links the synthetic matrices to the portfolio volatility spread of hierarchical risk parity vs. equal risk contribution	Papenbrock et al., 2021
Identification of stock crisis variables	GBDT	Robust identification of the most important variables planning stock market crises, and a local explanation of the crisis probability at each date through a features attribution	Benhamou et al., 2021a

### 7.2 Case study of XAI application to portfolio management

XAI has emerged as a crucial component in the field of portfolio management. Given the complexity of financial markets and the myriad factors affecting asset prices, investment decisions are increasingly informed by sophisticated machine learning models often seen as “black boxes” due to their inherent complexity and lack of interpretability. This lack of transparency can be problematic in a portfolio management context where understanding the reasons behind predictions is vital for risk management and strategic decision-making. XAI comes into play here by providing insights into what the model has learned and how it makes decisions. It provides a way to unravel the complex web of calculations machine learning models perform, intense learning ones.

Specifically, XAI techniques are essential for portfolio allocation decisions and for predicting returns using machine learning. Fund managers need to know why certain assets are favored by the model when allocating resources among various assets in a portfolio. This understanding helps identify the critical drivers behind portfolio allocation, improve trust in the model's decisions, and make more informed allocation adjustments. Regarding return predictions, explainability can reveal the model's sensitivity to certain features. It can also help ensure regulatory compliance, as financial regulators often require firms to explain their algorithmic decision-making processes. Thus, in portfolio choices and return predictions, XAI is critical in enhancing transparency, promoting trust, and ensuring better governance when using AI in portfolio management.

## 8 Discussion

The magic of AI is its ability to process big data at speed and accuracy that is not achievable by humans or conventional methods, to learn from the data and its mistakes, and to evolve and cope with high-complexity tasks. The primary role of the paper is to systematically review the existing state-of-the-art AI approaches used for asset allocation in each step of the portfolio management framework. However, the rapid surge in performance of sophisticated AI-powered systems turned them into black-box models, raising uncertainties about how the decisions are generated (Linardatos et al., 2021). This perfectly explains why the adoption of AI in finance still struggles, as high-speed investment decision-making should satisfy requirements such as reliability/soundness, accountability, transparency, fairness and ethics, which have been declared as the critical determinants of trustworthy solutions (Prenio and Yong, 2021). As a result, XAI has gained increased attention as a means (1) to develop more explainable models while preserving a high level of learning performance and (2) to enable humans to understand how the model works at its core, appropriately trust, and embrace the benefits of AI as artificially intelligent advisor or autonomous system (Arrieta et al., 2020).

The field of XAI, being comparatively new, is a rapidly growing body of research and, therefore, is still very fragmented. On the one hand, as an alternative to transparent/interpretable models, we observe a continuous development of different *post-hoc* explainability approaches and their extensions, which broadly could be categorized into model-agnostic and model-specific (PWc, 2018; Arrieta et al., 2020). On the other hand, in parallel, the conceptual frameworks, standards, and requirements are being published in their early stages by different bodies of the financial market. For example, in response to AI-powered risk, the draft EU regulatory framework on AI named the AI Act, was published in April 2021 (The European Commission, 2021). Under the proposed AI Act, a technology-neutral definition of AI system is established, and a risk-based classification is laid down, introducing prohibited AI systems, high-risk AI systems, AI systems subject to transparency requirements, and low-risk AI systems. This implies that different requirements and obligations will be applied accordingly, but this has not been settled definitively. Based on its current version, it may be concluded that the intelligent AI-powered portfolio management system is assigned to a low-risk case. As another example, in 2022, the Bank of England and the Financial Conduct Authority published their report on Artificial Intelligence Public-Private Forum (The Bank of England and the Financial Conduct Authority, 2022) summarizing the dialogue between the public sector, the private sector, and academia on AI. In the context of the use of AI in savings and investment management, the authors posed a potential AI-powered risk on markets in case AI becomes more widely used in institutional fund products, as this could lead to a “herd” behavior due to the similar data and models used or through concentrations in the networks used to transfer data and models, which ultimately affect consumers, firms, and the financial system. It has been envisioned as future steps that an industry body for practitioners could build trustworthy AI. At the same time, the regulators should support the innovation and accommodation of AI by clarifying how existing policies and regulations and policies apply to AI. Comparatively, in the report of IOSCO on the use of AI and ML by market intermediaries and asset managers (The Board of the International Organization of Securities Commissions, 2021), the areas such as (1) governance and oversight, (2) algorithm development, testing and ongoing monitoring, (3) data quality and bias, (4) transparency, and explainability, (5) outsourcing, and (6) ethical concerns were highlighted, where potential risks and harms may arise to AI-powered product development. Based on the responses received, the guidance consisting of six measures that reflect expected standards of conduct by market intermediaries and asset managers using AI is provided. Moreover, as the use of AI evolves in line with technological advances, the regulatory framework will need to be updated in tandem to address new emerging risks. And finally, any data-specific solution should inevitably align with GDPR principles (The European Commission, 2016, 2019). According to Tang (2019), adopting AI may violate GDPR provisions concerning two data-related rights, automated decision-making and the right to erasure, and two GDPR principles, i.e., transparency and data minimization.

As market intermediaries and institutional asset managers are developing solutions and products based on AI innovations, there is an increasing need to create and use AI-specific standards, which are now at a very conceptual level. Investment businesses are generally regulated at four levels: financial service providers as companies, product structure, product sales, and markets. As AI standardization is still in its early stages, we may only hypothesize that we expect an update of transparency regulation at the product level. For example, already today, a UCITS fund “should not invest in financial indices whose methodology for the selection and the rebalancing of the components is not based on a set of pre-determined rules and objective criteria” according to ESMA guidelines (ESMA, 2014, par. 58). This excludes dynamically learned rules from an AI system to create an index suitable as a reference underlying. Furthermore, we expect updated regulation at the market level. The FCA report (FCA, 2018) reviews excellent and bad algorithmic trading practices. MiFID II addresses algorithmic trading, but ESMA (2021) points out “that the use of algorithms which only serve to inform a trader of a particular investment opportunity is not considered as algorithmic trading, provided that the execution is not algorithmic”. Regulators prefer human oversight and judgement to fully automated systems. We speculate future regulation might require explainable AI concepts to enable humans to realize this oversight better.

So, it seems that much work still has to be done to retain control and safety, maintain trust and ethics, and comply with accountability and regulation. Ultimately, it may be concluded that the success of AI applications for portfolio management, and more generally, the products and services provided in the financial sector, will be only guaranteed if all these AI-related principles are harmonized.

## Author contributions

KS: Writing—original draft, Writing—review & editing. PS: Writing—original draft, Writing—review & editing. CS: Writing—original draft, Writing—review & editing. LL: Writing—original draft, Writing—review & editing. MM: Writing—original draft, Writing—review & editing. PL: Writing—original draft, Writing—review & editing. AK: Writing—original draft, Writing—review & editing. CT: Writing—original draft, Writing—review & editing. BO: Writing—original draft, Writing—review & editing. JC: Writing—original draft, Writing—review & editing.
